# FlyOde - a platform for community curation and interactive visualization of dynamic gene regulatory networks in *Drosophila* eye development

**DOI:** 10.12688/f1000research.7556.1

**Published:** 2015-12-21

**Authors:** Stefan A. Koestler, Begum Alaybeyoglu, Christian X. Weichenberger, Arzu Celik

**Affiliations:** 1 Department of Molecular Biology and Genetics, Bogazici University, Istanbul, 34342, Turkey; 2 Department of Chemical Engineering, Bogazici University, Istanbul, 34342, Turkey; 3 Life Sciences Center, Bogazici University, Istanbul, 34342, Turkey; 4 Center for Biomedicine, European Academy of Bozen/Bolzano (EURAC), (Affiliated to the University of Lübeck, Lübeck, Germany), Bozen/Bolzano, 39100, Italy

**Keywords:** Drosophila development, gene regulatory network, protein interaction network, visualization, web-tool, database, community annotation, ontology

## Abstract

**Motivation:** Understanding the regulatory mechanisms governing eye development of the model organism *Drosophila melanogaster (D. m.)* requires structured knowledge of the involved genes and proteins, their interactions, and dynamic expression patterns. Especially the latter information is however to a large extent scattered throughout the literature.

**Results:** FlyOde is an online platform for the systematic assembly of data on *D. m.* eye development. It consists of data on eye development obtained from the literature, and a web interface for users to interactively display these data as a gene regulatory network. Our manual curation process provides high standard structured data, following a specifically designed ontology. Visualization of gene interactions provides an overview of network topology, and filtering according to user-defined expression patterns makes it a versatile tool for daily tasks, as demonstrated by usage examples. Users are encouraged to submit additional data via a simple online form.

## Introduction

Developmental biology is the study of processes that generate an entire organism from a single cell. A central question in this field is how differentiation produces specific cell types from pluripotent precursors. *Drosophila melanogaster (D. m.)* serves as a suitable and well-established model organism to address this question for numerous reasons including a short generation time, the multitude of available genetic methods, and its orthology shared with vertebrates ( Reiter *et al.*, 2001). The *D. m.* eye allows the study of morphological rearrangements as well as differentiation of non-neuronal and neuronal cell types like photoreceptors (PRs) on the single cell level ( Thomas & Wassarman, 1999).

Understanding cell differentiation requires knowledge of the involved genes, their temporally varying (dynamic) expression patterns, and interactions. Interaction data from different sources are accessible through e.g. Biogrid, Intact, String, and REDfly ( Chatr-Aryamontri *et al.*, 2015; Gallo *et al.*, 2011; Orchard *et al.*, 2014; Szklarczyk *et al.*, 2015) and database collections, e.g. iRefindex or mentha ( Calderone *et al.*, 2013) ( Razick *et al.*, 2008). Expression data is mostly provided on embryonic development or organ systems, e.g. by FlyBase ( dos Santos *et al.*, 2015) and the Berkeley Drosophila Genome Project (BDGP) ( Hammonds *et al.*, 2013), but the coverage and precision of expression pattern annotation on the cellular level and the temporal resolution at later stages, e.g. larva and pupa, are limited (see Supplementary Material). On the other hand, a wealth of expression pattern data on these levels is contained in publications (e.g. ( Potier *et al.*, 2014)). Systematic use of these data requires their structured assembly through an extensive curation effort. Since automated curation is prone to errors ( Mao *et al.*, 2014), information must be extracted manually from the literature by experts, who can critically interpret the respective types of data like micrographs or expression profiles ( Tomancak *et al.*, 2007) and convert these to machine-readable data for further computer-based analyses.

We have thus developed FlyOde, an online hub for the community-driven systematic assembly of data on *D. m.* eye development. FlyOde is a web interface for interactive exploration of gene and protein relationships by combining visualization of the curated gene regulatory network with filters specific to fly development. FlyOde is built on an ontology-driven curation process that stores data in a specifically formatted text file, which can easily be enriched and extended upon arrival of new data.

## Implementation

### Data

**Figure 1.**
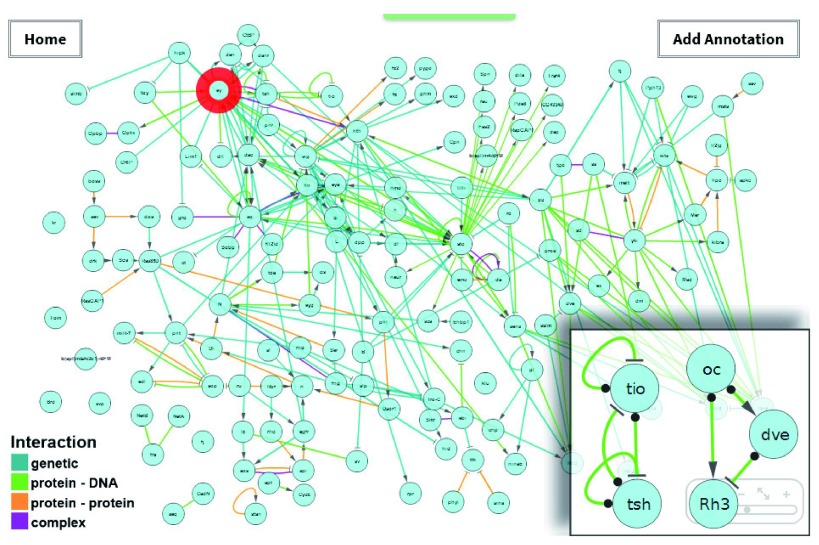
The FlyOde interface displaying the eye developmental network. The red circle indicates the *eyeless* gene (see Example 1). The inset shows selected interactions containing network motifs: negative autoregulation and feedback loop (left) and an incoherent feed forward loop (right).

**Figure 2.**
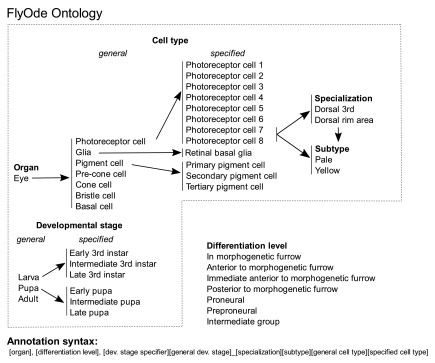
The FlyOde eye-ontology. Hierarchical representation of controlled vocabulary for the gene annotation with dynamic expression patterns is shown. In the annotation syntax terms for cell type and developmental stage are directly linked. Each term-combination represents expression in a specific cell type and developmental stage. As many comma-separated term combinations as needed to describe the expression pattern are given. During retinal development of *Drosophila,* cells at consecutive stages of differentiation coexist at the same developmental stage of the organism. The morphogenetic furrow marks the initiation of differentiation and moves across the eye precursor organ, the eye imaginal disc. Thus, the expression pattern is also defined by the localization relative to the morphogenetic furrow. This and additional terms that are common in the field are assigned to what we call “differentiation level”.

A directed gene interaction network representing eye development of *D. m.* from the third instar larva to the adult with focus on PR differentiation was constructed using Cytoscape ( Shannon *et al.*, 2003), based on data extracted manually from 77 publications ( Figure 1). Currently, the network contains 146 nodes representing genes/proteins, and 284 edges representing activating or inhibiting genetic, protein-protein, or protein-DNA interactions. The layout is generated manually and organized to approximately represent developmental time along the horizontal axis, beginning with early third instar larval stage on the left, and network hierarchy along the vertical axis with master regulators (as defined by ( Chan & Kyba, 2013)) placed towards the top. Nodes are associated with their FlyBase symbol, name, alternative names, FlyBase link, dynamic expression pattern, phenotypes, the terms for each of the three Gene Ontologies (GO) ( Ashburner *et al.*, 2000), and the literature references. GO annotations were added using Cytoscape. Expression pattern annotation follows a specifically developed ontology which links developmental stage and cell type ( Figure 2). We support and encourage annotation from the community to continually extend the dataset.

### Web application

The interactive JavaScript based web application renders the manually curated network, which is embedded using Cytoscape Web ( Lopes *et al.*, 2010). Genes are represented as nodes and gene (or protein) interactions are visualized as directed edges. Nodes and edges have been annotated during the curation process (see previous section), and this information is displayed when an item is selected. For the selection of nodes a module for searching gene symbols, names, alternative names, and GO-terms is provided. Additionally, the network can be filtered according to gene expression patterns using dropdown menus whose structure follows the FlyOde ontology ( Figure 2). Filters can be combined with the Boolean operators AND, OR, and NOT. Extensions to the network can be submitted to us via a community curation form.

## Characterization

To characterize the content of the current FlyOde network GO enrichment analysis was performed with the ClueGO app in Cytoscape ( Bindea *et al.*, 2009). It shows that terms correlated with development and morphogenesis, pattern formation and polarity, apoptosis, mitosis, and regulation of transcription are highly over-represented, as expected (see Supplementary Materials and Supplementary Figure 1 and Supplementary Figure 2).

To evaluate the information that can be obtained from FlyOde the FlyOde filter function was compared with the FlyBase QueryBuilder. When queried for general terms like “larva and PR”, FlyBase gave more gene hits than FlyOde, but when increasingly specifying the developmental stage and cell type, like “pupa and dorsal rim area R8 (photoreceptor cell 8)”, more genes were found with FlyOde as compared to FlyBase. This shows that FlyOde already stands out in defining gene expression in a specific cell type and during a specific developmental stage of PR differentiation (see Supplementary Materials and Supplementary Figure 3).

To get an idea of the basic mechanisms represented in FlyOde, the occurrence of network motifs was analysed manually in Cytoscape. Motifs commonly found during development of organisms, like autoregulation, feedback and feed forward loops were observed. This indicates that FlyOde at least partially displays the connectivity and level of detail to qualify for representation and detailed analysis of developmental processes (see Figure 1 and Supplementary Materials, Supplementary Figure 4 and Supplementary Table) ( Alon, 2007; Davidson, 2010).

## Use cases

### Example 1: Obtaining information on a specific gene

In this example we use the search functionality to explore a specific gene in the dataset. In order to do so, *Pax6* is entered into the search box. This gene is known under that name as an important regulator of development in many organisms ( van Heyningen, 2002). However, the official name in *D. m*. is *eyeless*. Due to its annotation with alternative names it is found despite the search for the unofficial name *Pax6* and highlighted in the network ( Figure 1). Its position on the left side of the graph indicates that it is mainly expressed early during eye development. In the web interface additional information is displayed in a content related text field below the graph, which we designate “report panel”. A closer look at the expression pattern displayed there shows that it is expressed anterior to the morphogenetic furrow, in all photoreceptors in the early third instar larva, and in outer photoreceptors in the late pupa and adult. The top position in the hierarchy indicates that it is a master regulator. This is supported by its high number of interactions, and by the phenotypes, which are given in the report panel ( Chan & Kyba, 2013). We also find that GO annotates *eyeless* as a transcription factor, and that it is involved in developmental processes of other organs. Finally, the literature references and the FlyBase link can be followed for further information.

### Example 2: Which genes can be used as markers for PR R8 in the intermediate pupa?

Here we apply filter combinations to display genes that are expressed in R8, but not in any other PR in the intermediate pupa to obtain candidate markers for R8 in immunostaining and cell sorting experiments. In the dropdown menus we choose “pupa”, “intermediate pupa”, “photoreceptor cell”, and “R8”, respectively. We add another filter line with the Boolean “NOT”, and in the dropdown menus select “intermediate pupa”, “photoreceptor cell”, “R7”, and another filter line with the Boolean “NOT”, “intermediate pupa”, and “at least one outer PR”, which in combination with “NOT” means “no outer PR”. All nodes are removed except for *scabrous* and *senseless*, which are therefore candidates for being R8 markers. The literature references for these two markers provide a starting point for future experimental studies.

More examples are provided in the tutorial at http://flyode.boun.edu.tr/quickguide.html.

## Discussion

Here we have presented FlyOde, which provides a platform for combining published data on gene regulatory networks (GRNs) of *Drosophila* organ development. As a starting point, we have equipped FlyOde with extensive GRN data for *D. m.* eye development, such that the web interface serves as a versatile tool for everyday tasks a fly researcher encounters.

FlyOde delivers high quality data standards by manual curation. We expect to achieve efficient data collection by distributing the annotation workload among community members with minimal effort for the individual contributors, who only need to submit a simple form to add new nodes, interactions, or annotation. In return, they directly profit from the improved tool by linking their data of interest with the shared knowledge.

We are constantly improving the ontology and web application, in parallel to ongoing data curation and dataset extension. FlyOde will be expanded to include other organs with the ultimate goal to compare their GRNs.

Future work will profit from the obtained data by constraining network inference from gene expression data ( Hecker *et al.*, 2009). Another anticipated approach is to assign quantitative expression data to the established network to facilitate mathematical modelling ( Graham *et al.*, 2010).

We envision FlyOde as a companion that guides researchers through developmental processes, for example while studying a paper, and believe that the community-curated dataset and its analysis will add significant knowledge to developmental biology.

## Data availability

The data referenced by this article are under copyright with the following copyright statement: Copyright: © 2015 Koestler SA et al.

Data associated with the article are available under the terms of the Creative Commons Attribution Licence, which permits unrestricted use, distribution, and reproduction in any medium, provided the original data is properly cited. 

FlyOde, including all network data, annotations and their corresponding references can be freely accessed via the web-application at http://flyode.boun.edu.tr/.

## Software availability

### Software access

The source code for the web application can be downloaded from Github ( https://github.com/begum-alaybeyoglu/FlyOde).

### Source code as at the time of publication

https://github.com/F1000Research/FlyOde

### Archived source code as at the time of publication

http://dx.doi.org/10.5281/zenodo.35227

### Software license

The FlyOde web application is released under the MIT License.

## Supplementary Material

**Supplementary Figure 1.**

GO-term enrichment analysis with ClueGO.

**Analysis and characterization of the FlyOde network.**
