# Supplementary material for: FlyOde - a platform for community curation and interactive visualization of dynamic gene regulatory networks in Drosophila eye development
Source: F1000Res. 2015 Dec 21;4:1484. [Version 1] doi: 10.12688/f1000research.7556.1 (PMC4786896; doi:10.12688/f1000research.7556.1)
Supplement: Supplementary file 1 [file f1000research-4-8136-s0001.tgz › 6b97fde5-6cd1-480a-bd60-0713c234bd90.pdf]

**Supplementary Figure 1**: See following pages. ClueGO results. The bar graph displays the percentage of FlyOde genes for each term relative to the reference. The absolute numbers of the respective FlyOde genes are shown next to the bars. Colours indicate term groups shared by genes enriched in FlyOde. Specific terms mentioned in the text are highlighted in red.

# Supplementary Figure 2

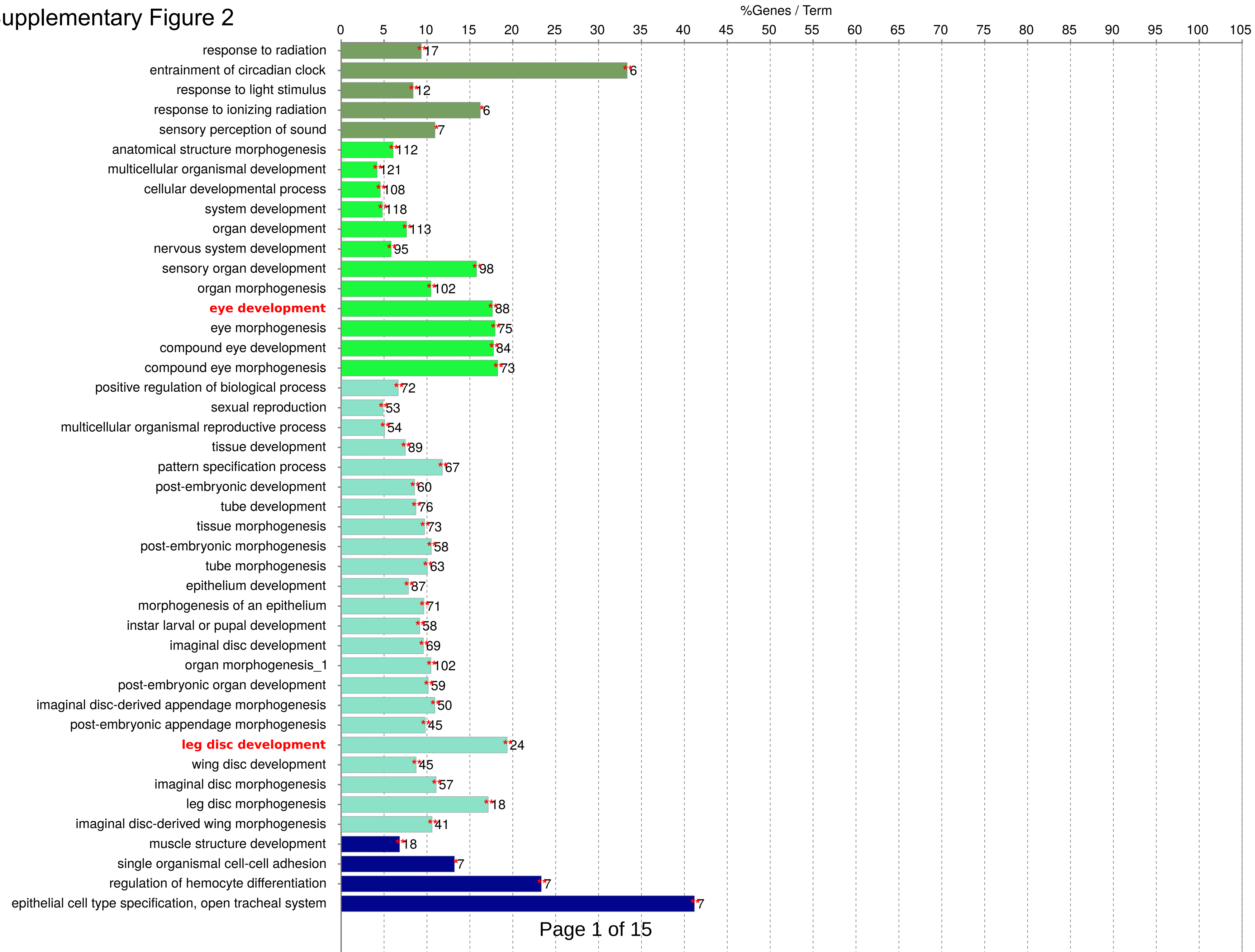

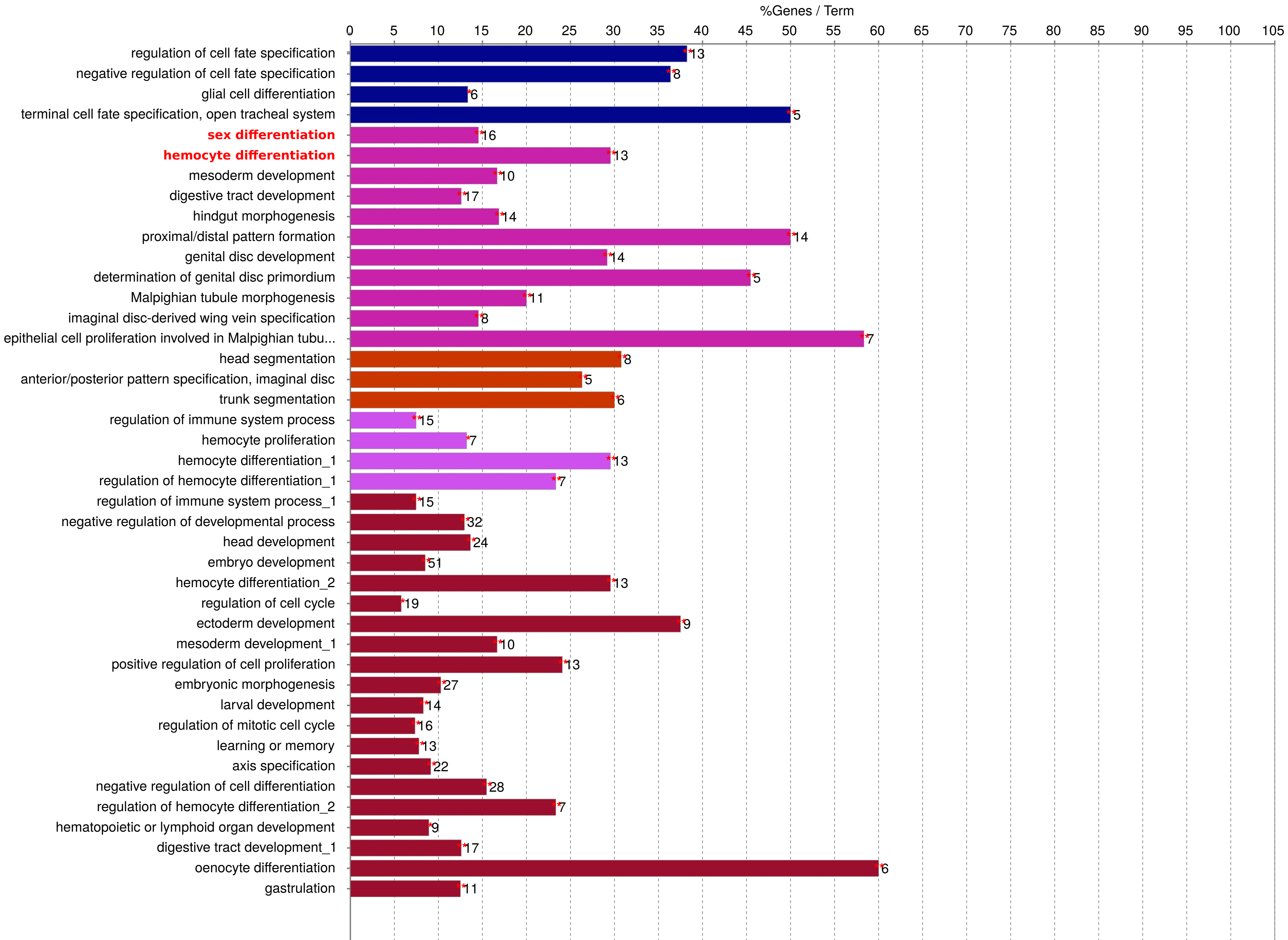
%Genes / Term
0 5 10 15 20 25 30 35 40 45 50 55 60 65 70 75 80 85 90 95 100 105
regulation of cell fate specification **13
negative regulation of cell fate specification **8
glial cell differentiation *6
terminal cell fate specification, open tracheal system **5
sex differentiation **16
hemocyte differentiation **13
mesoderm development **10
digestive tract development **17
hindgut morphogenesis **14
proximal/distal pattern formation **14
genital disc development **14
determination of genital disc primordium **5
Malpighian tubule morphogenesis **11
imaginal disc-derived wing vein specification **8
epithelial cell proliferation involved in Malpighian tubu... **7
head segmentation *8
anterior/posterior pattern specification, imaginal disc *5
trunk segmentation *6
regulation of immune system process **15
hemocyte proliferation *7
hemocyte differentiation_1 **13
regulation of hemocyte differentiation_1 **7
regulation of immune system process_1 *15
negative regulation of developmental process **32
head development *24
embryo development *51
hemocyte differentiation_2 **13
regulation of cell cycle *19
ectoderm development *9
mesoderm development_1 **10
positive regulation of cell proliferation *13
embryonic morphogenesis *27
larval development *14
regulation of mitotic cell cycle *16
learning or memory *13
axis specification **22
negative regulation of cell differentiation *28
regulation of hemocyte differentiation_2 *7
hematopoietic or lymphoid organ development *9
digestive tract development_1 **17
oenocyte differentiation *6
gastrulation **11

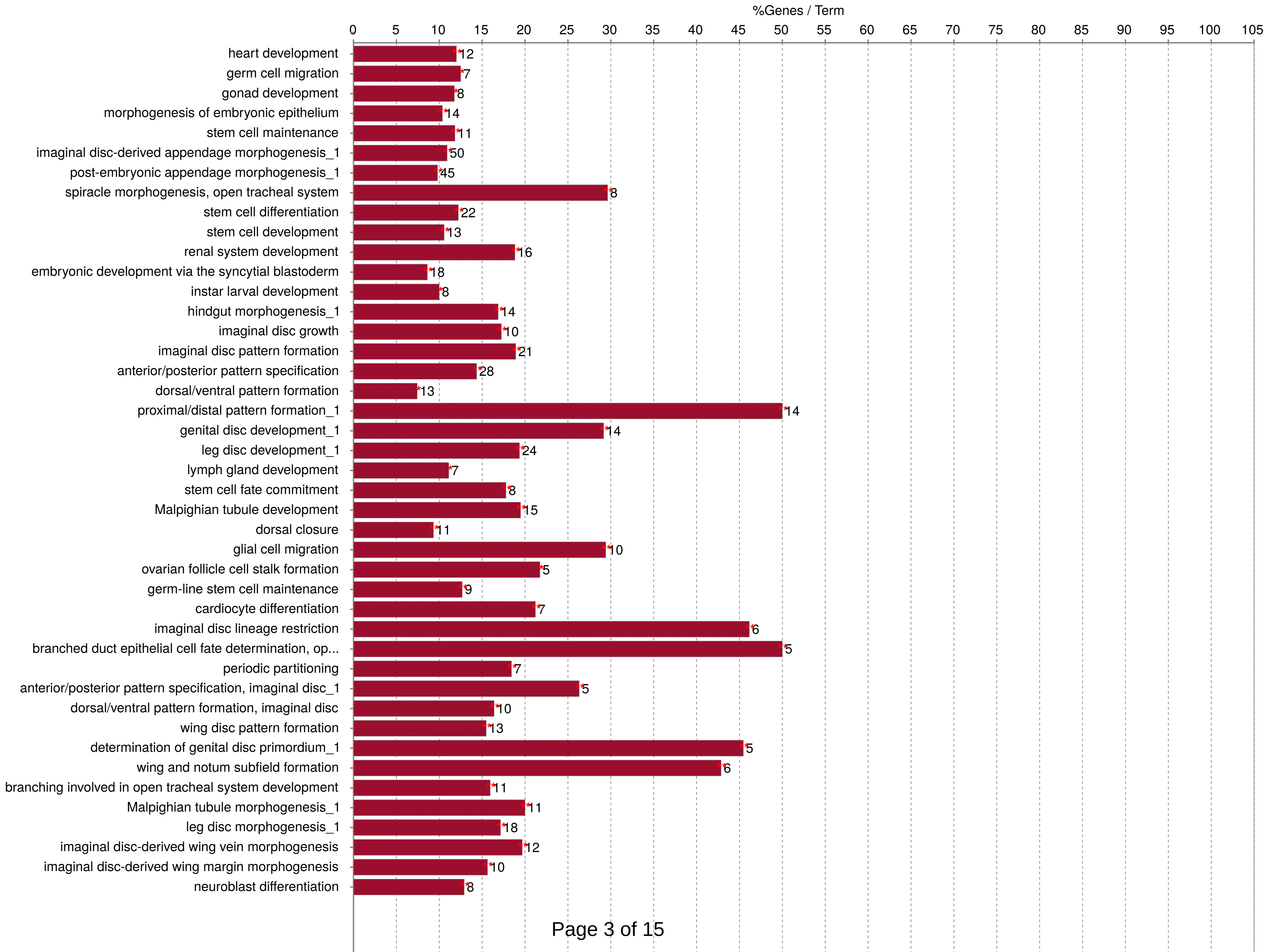
%Genes / Term
0
5
10
15
20
25
30
35
40
45
50
55
60
65
70
75
80
85
90
95
100
105
heart development
*12
germ cell migration
*7
gonad development
*8
morphogenesis of embryonic epithelium
*14
stem cell maintenance
*11
imaginal disc-derived appendage morphogenesis_1
*50
post-embryonic appendage morphogenesis_1
*45
spiracle morphogenesis, open tracheal system
*8
stem cell differentiation
*22
stem cell development
*13
renal system development
*16
embryonic development via the syncytial blastoderm
*18
instar larval development
*8
hindgut morphogenesis_1
*14
imaginal disc growth
*10
imaginal disc pattern formation
*21
anterior/posterior pattern specification
*28
dorsal/ventral pattern formation
*13
proximal/distal pattern formation_1
*14
genital disc development_1
*14
leg disc development_1
*24
lymph gland development
*7
stem cell fate commitment
*8
Malpighian tubule development
*15
dorsal closure
*11
glial cell migration
*10
ovarian follicle cell stalk formation
*5
germ-line stem cell maintenance
*9
cardiocyte differentiation
*7
imaginal disc lineage restriction
*6
branched duct epithelial cell fate determination, op...
*5
periodic partitioning
*7
anterior/posterior pattern specification, imaginal disc_1
*5
dorsal/ventral pattern formation, imaginal disc
*10
wing disc pattern formation
*13
determination of genital disc primordium_1
*5
wing and notum subfield formation
*6
branching involved in open tracheal system development
*11
Malpighian tubule morphogenesis_1
*11
leg disc morphogenesis_1
*18
imaginal disc-derived wing vein morphogenesis
*12
imaginal disc-derived wing margin morphogenesis
*10
neuroblast differentiation
*8

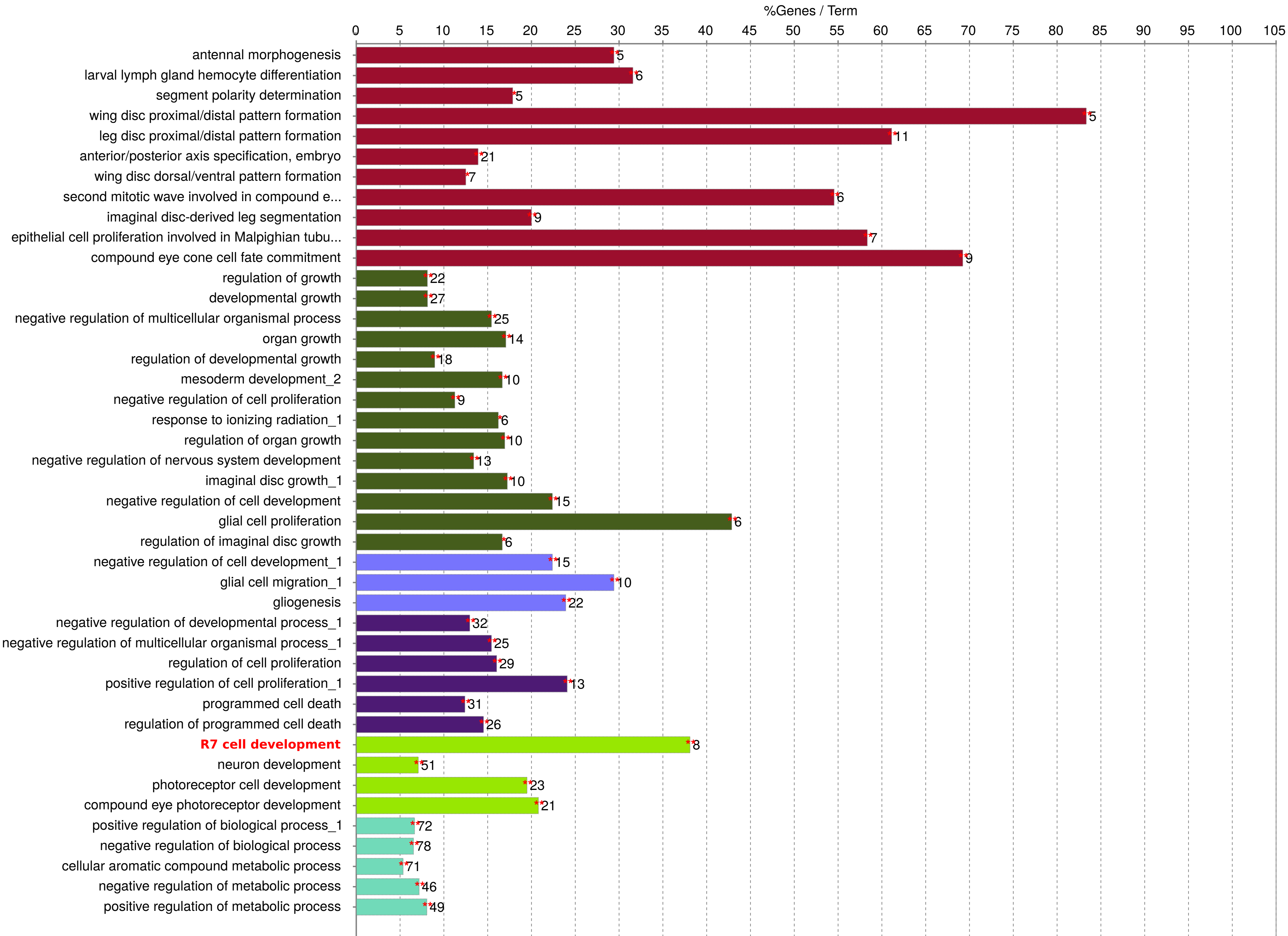
%Genes / Term
0
5
10
15
20
25
30
35
40
45
50
55
60
65
70
75
80
85
90
95
100
105
antennal morphogenesis
**5
larval lymph gland hemocyte differentiation
**6
segment polarity determination
*5
wing disc proximal/distal pattern formation
**5
leg disc proximal/distal pattern formation
**11
anterior/posterior axis specification, embryo
**21
wing disc dorsal/ventral pattern formation
*7
second mitotic wave involved in compound e...
**6
imaginal disc-derived leg segmentation
**9
epithelial cell proliferation involved in Malpighian tubu...
**7
compound eye cone cell fate commitment
**9
regulation of growth
**22
developmental growth
**27
negative regulation of multicellular organismal process
**25
organ growth
**14
regulation of developmental growth
**18
mesoderm development_2
**10
negative regulation of cell proliferation
**9
response to ionizing radiation_1
*6
regulation of organ growth
**10
negative regulation of nervous system development
**13
imaginal disc growth_1
**10
negative regulation of cell development
**15
glial cell proliferation
**6
regulation of imaginal disc growth
*6
negative regulation of cell development_1
**15
glial cell migration_1
**10
gliogenesis
**22
negative regulation of developmental process_1
**32
negative regulation of multicellular organismal process_1
**25
regulation of cell proliferation
**29
positive regulation of cell proliferation_1
**13
programmed cell death
**31
regulation of programmed cell death
**26
R7 cell development
**8
neuron development
**51
photoreceptor cell development
**23
compound eye photoreceptor development
**21
positive regulation of biological process_1
**72
negative regulation of biological process
**78
cellular aromatic compound metabolic process
**71
negative regulation of metabolic process
**46
positive regulation of metabolic process
**49

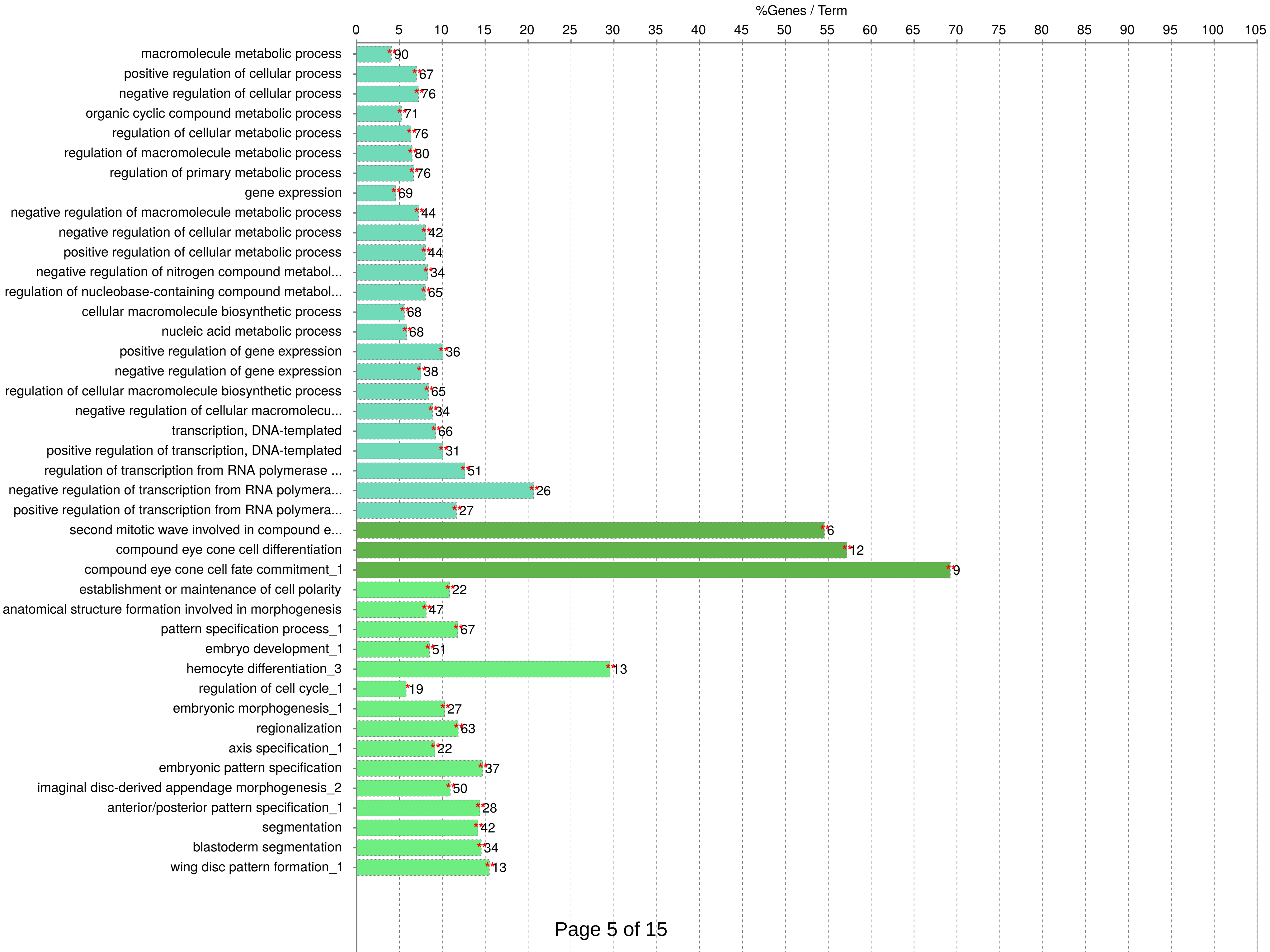

%Genes / Term
0
5
10
15
20
25
30
35
40
45
50
55
60
65
70
75
80
85
90
95
100
105
macromolecule metabolic process
**90
positive regulation of cellular process
**67
negative regulation of cellular process
**76
organic cyclic compound metabolic process
**71
regulation of cellular metabolic process
**76
regulation of macromolecule metabolic process
**80
regulation of primary metabolic process
**76
gene expression
**69
negative regulation of macromolecule metabolic process
**44
negative regulation of cellular metabolic process
**42
positive regulation of cellular metabolic process
**44
negative regulation of nitrogen compound metabol...
**34
regulation of nucleobase-containing compound metabol...
**65
cellular macromolecule biosynthetic process
**68
nucleic acid metabolic process
**68
positive regulation of gene expression
**36
negative regulation of gene expression
**38
regulation of cellular macromolecule biosynthetic process
**65
negative regulation of cellular macromolecu...
**34
transcription, DNA-templated
**66
positive regulation of transcription, DNA-templated
**31
regulation of transcription from RNA polymerase ...
**51
negative regulation of transcription from RNA polymera...
**26
positive regulation of transcription from RNA polymera...
**27
second mitotic wave involved in compound e...
**6
compound eye cone cell differentiation
**12
compound eye cone cell fate commitment_1
**9
establishment or maintenance of cell polarity
**22
anatomical structure formation involved in morphogenesis
**47
pattern specification process_1
**67
embryo development_1
**51
hemocyte differentiation_3
**13
regulation of cell cycle_1
*19
embryonic morphogenesis_1
**27
regionalization
**63
axis specification_1
**22
embryonic pattern specification
**37
imaginal disc-derived appendage morphogenesis_2
**50
anterior/posterior pattern specification_1
**28
segmentation
**42
blastoderm segmentation
**34
wing disc pattern formation_1
**13

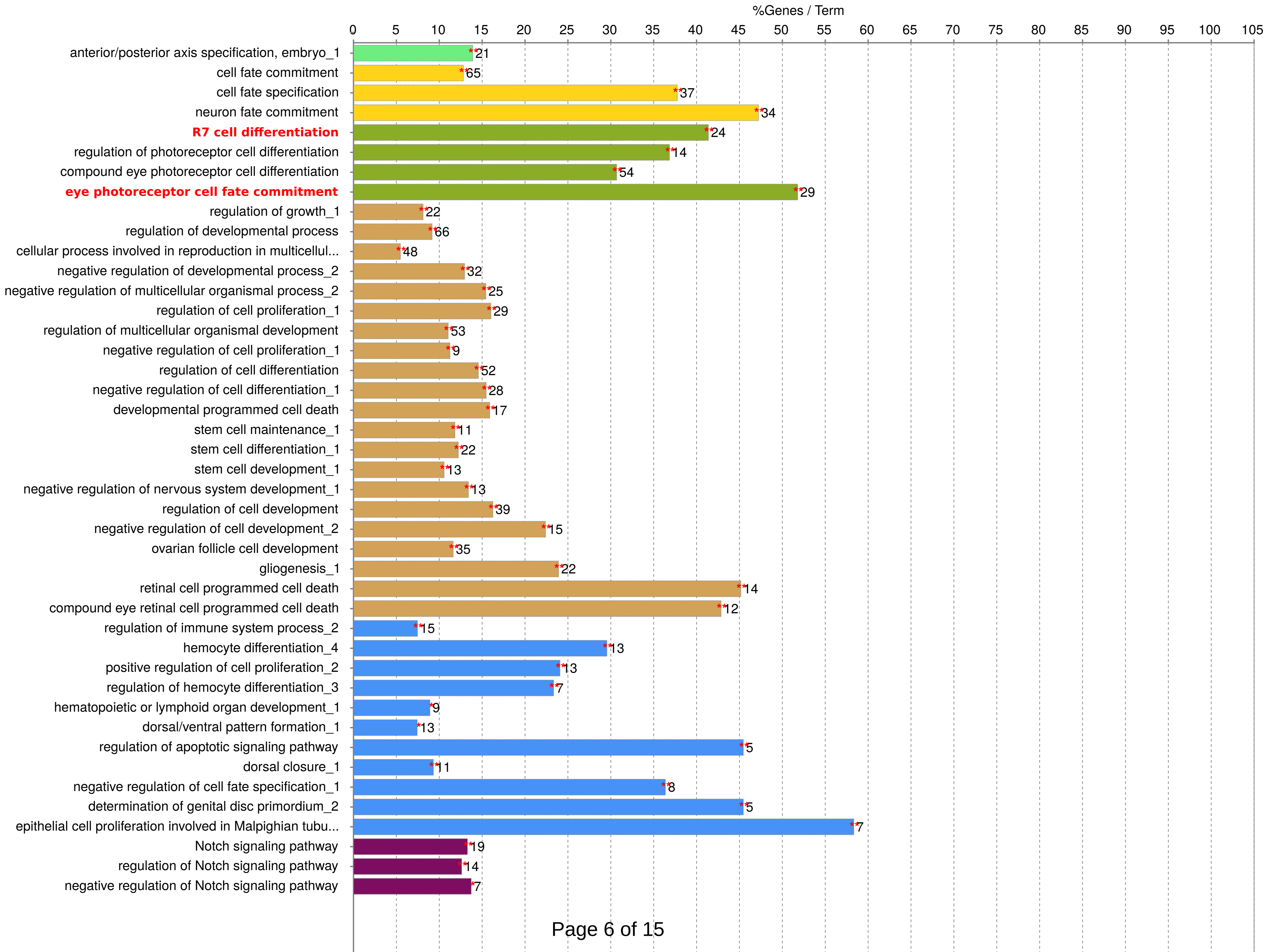

%Genes / Term
0
5
10
15
20
25
30
35
40
45
50
55
60
65
70
75
80
85
90
95
100
105
anterior/posterior axis specification, embryo_1
**21
cell fate commitment
**65
cell fate specification
**37
neuron fate commitment
**34
R7 cell differentiation
**24
regulation of photoreceptor cell differentiation
**14
compound eye photoreceptor cell differentiation
**54
eye photoreceptor cell fate commitment
**29
regulation of growth_1
**22
regulation of developmental process
**66
cellular process involved in reproduction in multicellul...
**48
negative regulation of developmental process_2
**32
negative regulation of multicellular organismal process_2
**25
regulation of cell proliferation_1
**29
regulation of multicellular organismal development
**53
negative regulation of cell proliferation_1
**9
regulation of cell differentiation
**52
negative regulation of cell differentiation_1
**28
developmental programmed cell death
**17
stem cell maintenance_1
**11
stem cell differentiation_1
**22
stem cell development_1
**13
negative regulation of nervous system development_1
**13
regulation of cell development
**39
negative regulation of cell development_2
**15
ovarian follicle cell development
**35
gliogenesis_1
**22
retinal cell programmed cell death
**14
compound eye retinal cell programmed cell death
**12
regulation of immune system process_2
**15
hemocyte differentiation_4
**13
positive regulation of cell proliferation_2
**13
regulation of hemocyte differentiation_3
**7
hematopoietic or lymphoid organ development_1
*9
dorsal/ventral pattern formation_1
*13
regulation of apoptotic signaling pathway
**5
dorsal closure_1
**11
negative regulation of cell fate specification_1
**8
determination of genital disc primordium_2
**5
epithelial cell proliferation involved in Malpighian tubu...
**7
Notch signaling pathway
**19
regulation of Notch signaling pathway
**14
negative regulation of Notch signaling pathway
*7

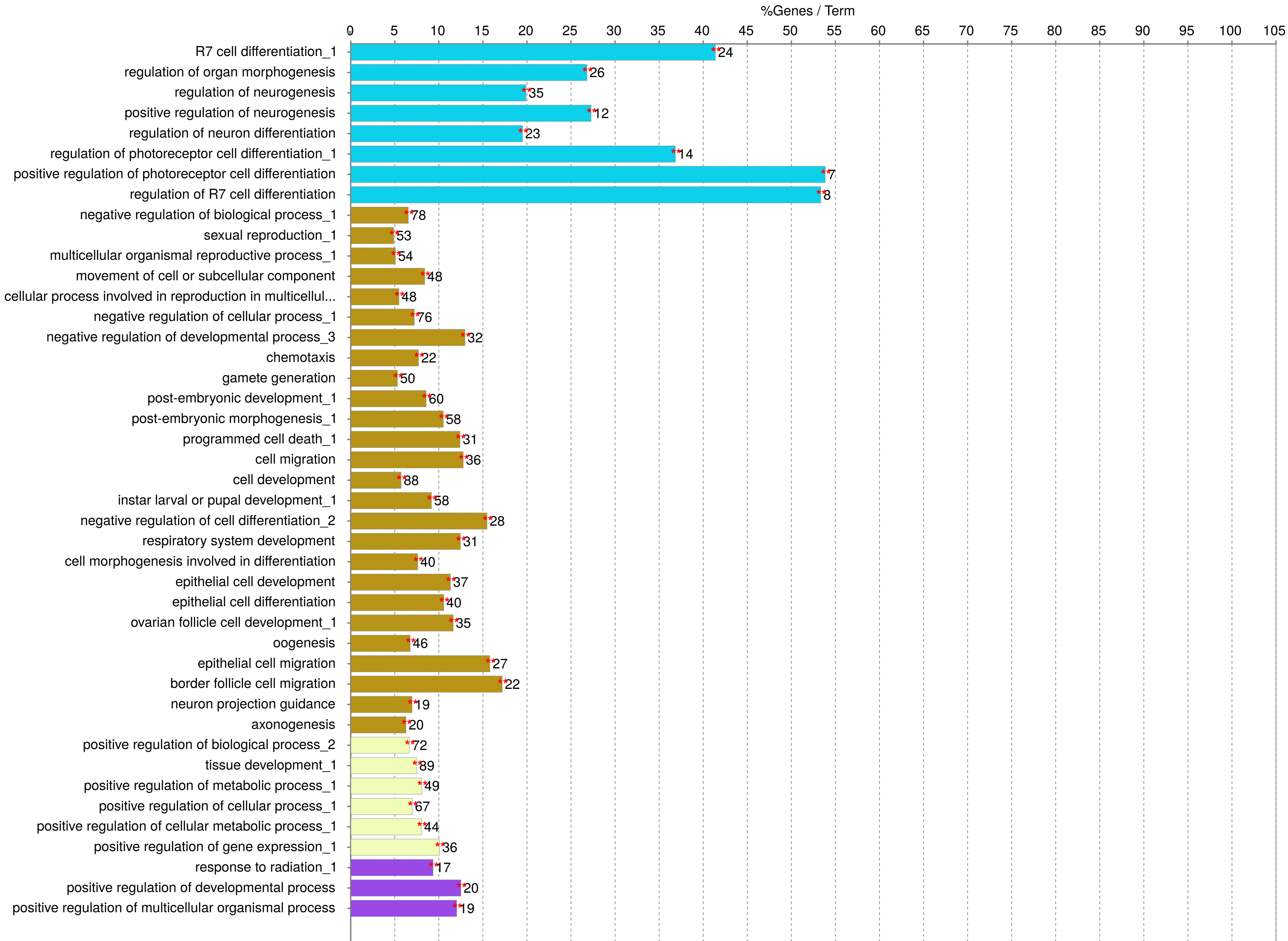
%Genes / Term
0
5
10
15
20
25
30
35
40
45
50
55
60
65
70
75
80
85
90
95
100
105
R7 cell differentiation_1
**24
regulation of organ morphogenesis
**26
regulation of neurogenesis
**35
positive regulation of neurogenesis
**12
regulation of neuron differentiation
**23
regulation of photoreceptor cell differentiation_1
**14
positive regulation of photoreceptor cell differentiation
**7
regulation of R7 cell differentiation
**8
negative regulation of biological process_1
**78
sexual reproduction_1
**53
multicellular organismal reproductive process_1
**54
movement of cell or subcellular component
**48
cellular process involved in reproduction in multicellul...
**48
negative regulation of cellular process_1
**76
negative regulation of developmental process_3
**32
chemotaxis
**22
gamete generation
**50
post-embryonic development_1
**60
post-embryonic morphogenesis_1
**58
programmed cell death_1
**31
cell migration
**36
cell development
**88
instar larval or pupal development_1
**58
negative regulation of cell differentiation_2
**28
respiratory system development
**31
cell morphogenesis involved in differentiation
**40
epithelial cell development
**37
epithelial cell differentiation
**40
ovarian follicle cell development_1
**35
oogenesis
**46
epithelial cell migration
**27
border follicle cell migration
**22
neuron projection guidance
**19
axonogenesis
**20
positive regulation of biological process_2
**72
tissue development_1
**89
positive regulation of metabolic process_1
**49
positive regulation of cellular process_1
**67
positive regulation of cellular metabolic process_1
**44
positive regulation of gene expression_1
**36
response to radiation_1
**17
positive regulation of developmental process
**20
positive regulation of multicellular organismal process
**19

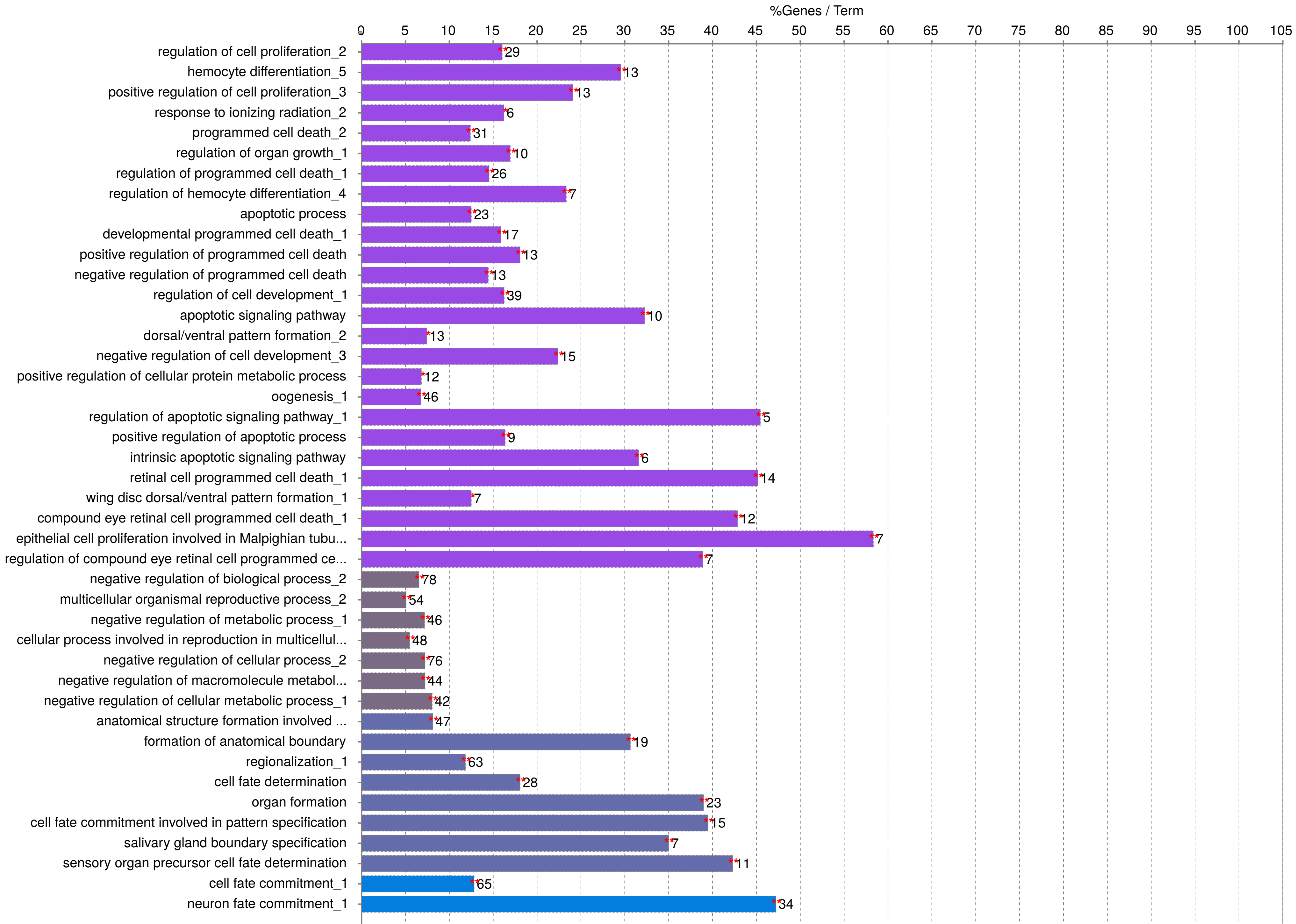
%Genes / Term
0
5
10
15
20
25
30
35
40
45
50
55
60
65
70
75
80
85
90
95
100
105
regulation of cell proliferation_2
**29
hemocyte differentiation_5
**13
positive regulation of cell proliferation_3
**13
response to ionizing radiation_2
*6
programmed cell death_2
**31
regulation of organ growth_1
**10
regulation of programmed cell death_1
**26
regulation of hemocyte differentiation_4
**7
apoptotic process
**23
developmental programmed cell death_1
**17
positive regulation of programmed cell death
**13
negative regulation of programmed cell death
**13
regulation of cell development_1
**39
apoptotic signaling pathway
**10
dorsal/ventral pattern formation_2
*13
negative regulation of cell development_3
**15
positive regulation of cellular protein metabolic process
*12
oogenesis_1
**46
regulation of apoptotic signaling pathway_1
**5
positive regulation of apoptotic process
**9
intrinsic apoptotic signaling pathway
**6
retinal cell programmed cell death_1
**14
wing disc dorsal/ventral pattern formation_1
*7
compound eye retinal cell programmed cell death_1
**12
epithelial cell proliferation involved in Malpighian tubu...
**7
regulation of compound eye retinal cell programmed ce...
**7
negative regulation of biological process_2
**78
multicellular organismal reproductive process_2
**54
negative regulation of metabolic process_1
**46
cellular process involved in reproduction in multicellul...
**48
negative regulation of cellular process_2
**76
negative regulation of macromolecule metabol...
**44
negative regulation of cellular metabolic process_1
**42
anatomical structure formation involved ...
**47
formation of anatomical boundary
**19
regionalization_1
**63
cell fate determination
**28
organ formation
**23
cell fate commitment involved in pattern specification
**15
salivary gland boundary specification
**7
sensory organ precursor cell fate determination
**11
cell fate commitment_1
**65
neuron fate commitment_1
**34

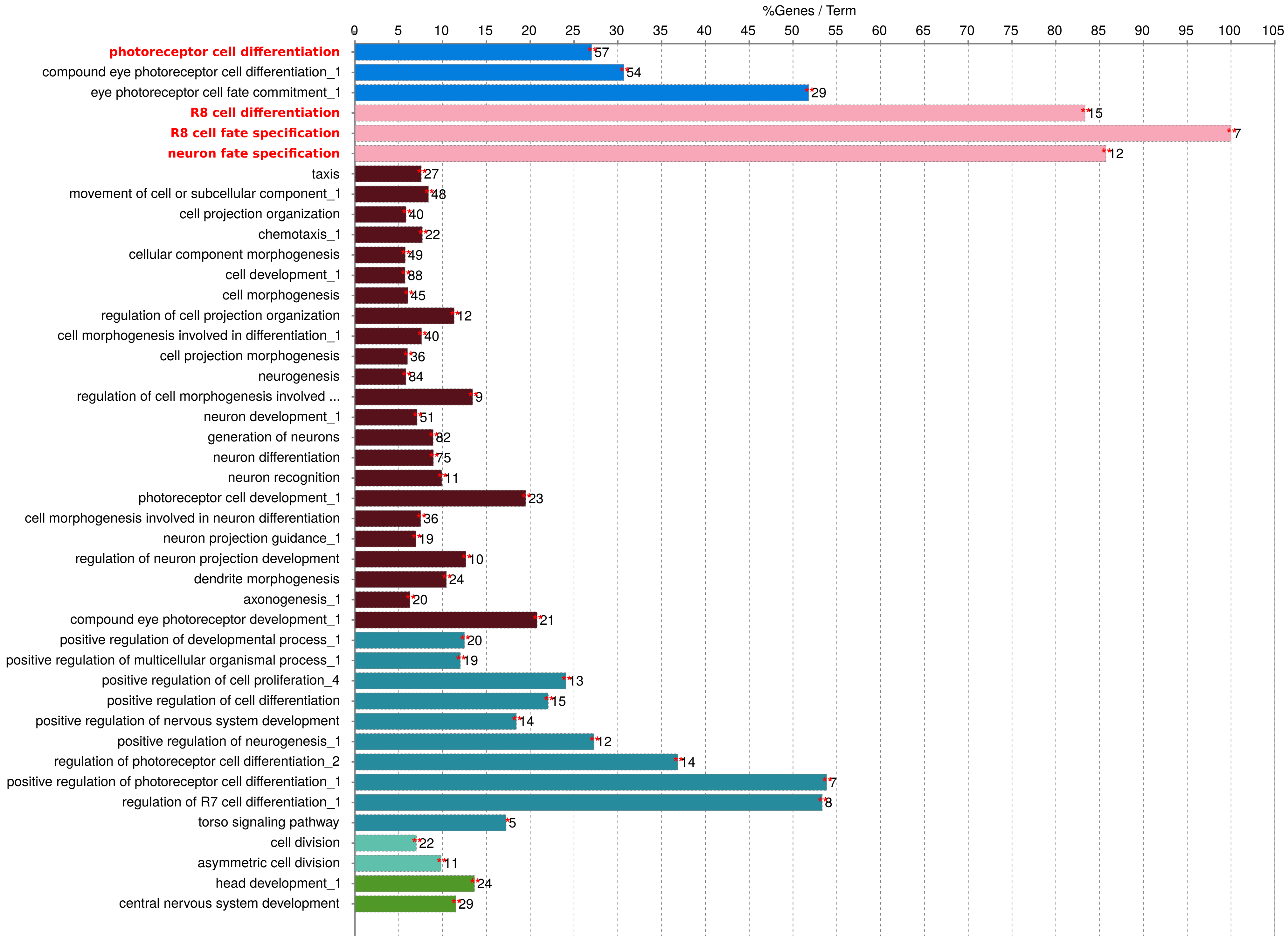
%Genes / Term
0 5 10 15 20 25 30 35 40 45 50 55 60 65 70 75 80 85 90 95 100 105
photoreceptor cell differentiation 57
compound eye photoreceptor cell differentiation_1 54
eye photoreceptor cell fate commitment_1 29
R8 cell differentiation 15
R8 cell fate specification 7
neuron fate specification 12
taxis 27
movement of cell or subcellular component_1 48
cell projection organization 40
chemotaxis_1 22
cellular component morphogenesis 49
cell development_1 88
cell morphogenesis 45
regulation of cell projection organization 12
cell morphogenesis involved in differentiation_1 40
cell projection morphogenesis 36
neurogenesis 84
regulation of cell morphogenesis involved ... 9
neuron development_1 51
generation of neurons 82
neuron differentiation 75
neuron recognition 11
photoreceptor cell development_1 23
cell morphogenesis involved in neuron differentiation 36
neuron projection guidance_1 19
regulation of neuron projection development 10
dendrite morphogenesis 24
axonogenesis_1 20
compound eye photoreceptor development_1 21
positive regulation of developmental process_1 20
positive regulation of multicellular organismal process_1 19
positive regulation of cell proliferation_4 13
positive regulation of cell differentiation 15
positive regulation of nervous system development 14
positive regulation of neurogenesis_1 12
regulation of photoreceptor cell differentiation_2 14
positive regulation of photoreceptor cell differentiation_1 7
regulation of R7 cell differentiation_1 8
torso signaling pathway 5
cell division 22
asymmetric cell division 11
head development_1 24
central nervous system development 29

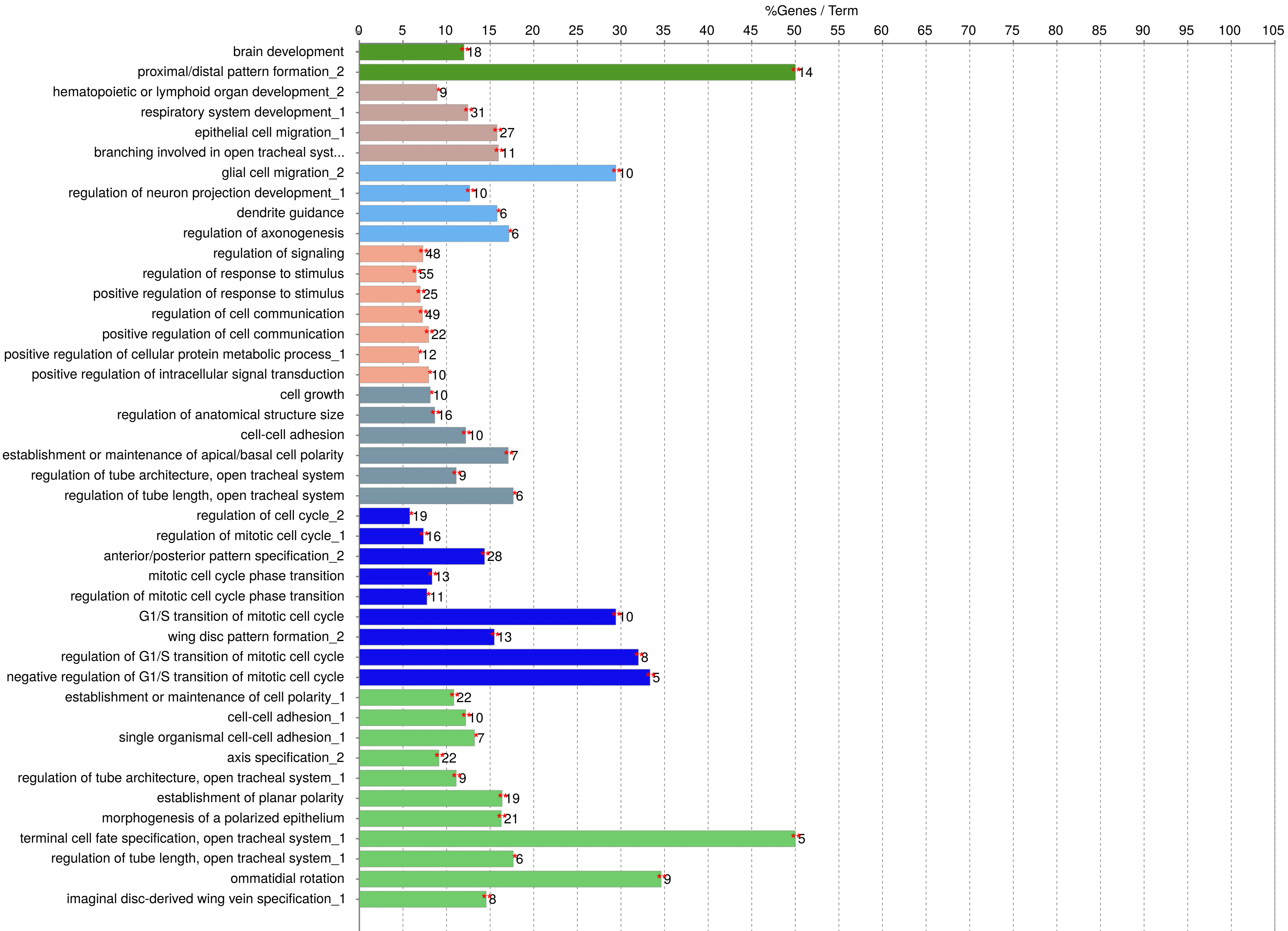
%Genes / Term
0 5 10 15 20 25 30 35 40 45 50 55 60 65 70 75 80 85 90 95 100 105
brain development **18
proximal/distal pattern formation_2 **14
hematopoietic or lymphoid organ development_2 *9
respiratory system development_1 **31
epithelial cell migration_1 **27
branching involved in open tracheal syst... **11
glial cell migration_2 **10
regulation of neuron projection development_1 **10
dendrite guidance *6
regulation of axonogenesis *6
regulation of signaling **48
regulation of response to stimulus **55
positive regulation of response to stimulus **25
regulation of cell communication **49
positive regulation of cell communication **22
positive regulation of cellular protein metabolic process_1 *12
positive regulation of intracellular signal transduction *10
cell growth *10
regulation of anatomical structure size **16
cell-cell adhesion **10
establishment or maintenance of apical/basal cell polarity **7
regulation of tube architecture, open tracheal system **9
regulation of tube length, open tracheal system *6
regulation of cell cycle_2 *19
regulation of mitotic cell cycle_1 **16
anterior/posterior pattern specification_2 **28
mitotic cell cycle phase transition **13
regulation of mitotic cell cycle phase transition *11
G1/S transition of mitotic cell cycle **10
wing disc pattern formation_2 **13
regulation of G1/S transition of mitotic cell cycle **8
negative regulation of G1/S transition of mitotic cell cycle **5
establishment or maintenance of cell polarity_1 **22
cell-cell adhesion_1 **10
single organismal cell-cell adhesion_1 *7
axis specification_2 **22
regulation of tube architecture, open tracheal system_1 **9
establishment of planar polarity **19
morphogenesis of a polarized epithelium **21
terminal cell fate specification, open tracheal system_1 **5
regulation of tube length, open tracheal system_1 *6
ommatidial rotation **9
imaginal disc-derived wing vein specification_1 **8

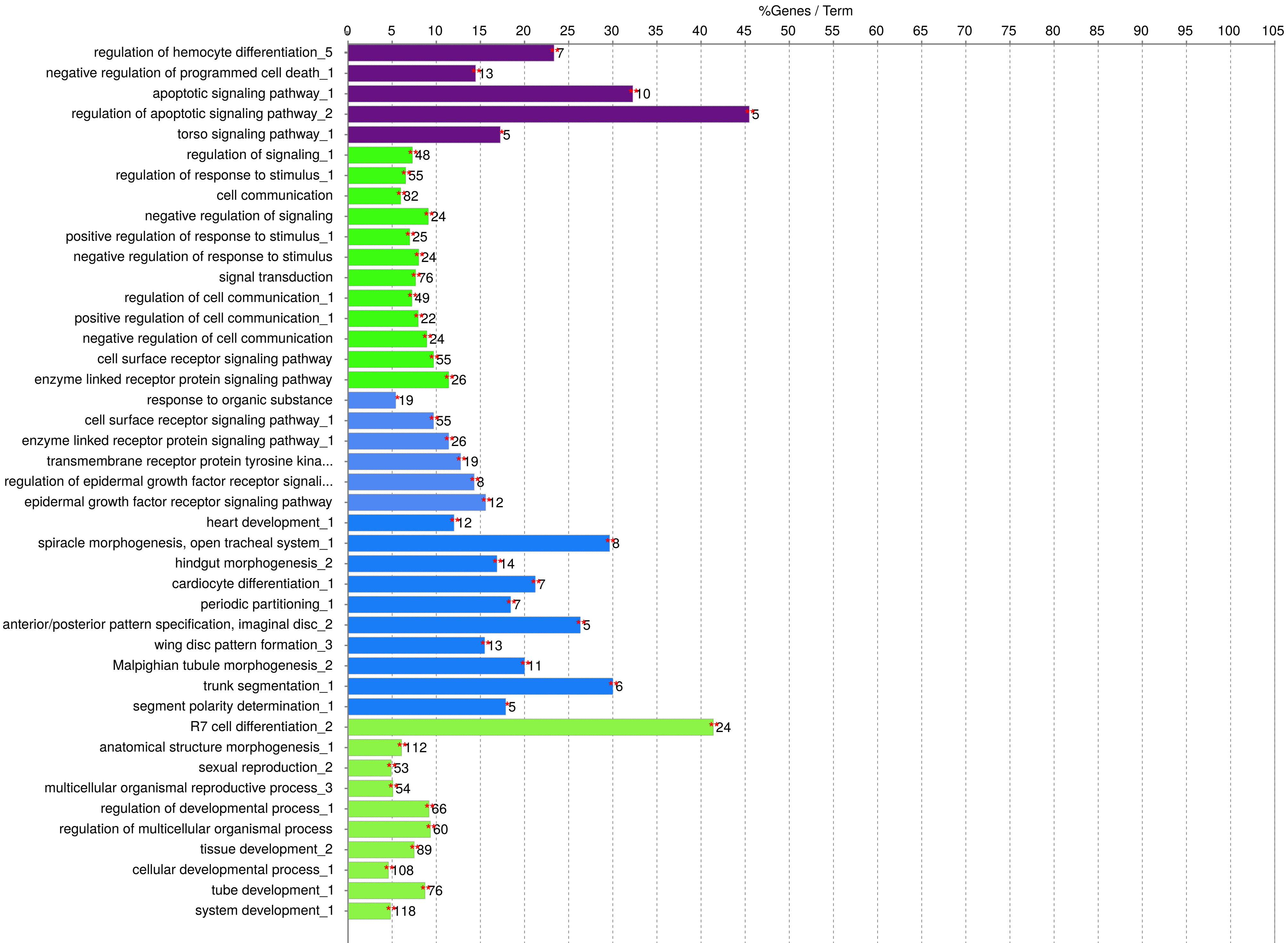
%Genes / Term
0 5 10 15 20 25 30 35 40 45 50 55 60 65 70 75 80 85 90 95 100 105
regulation of hemocyte differentiation_5 *7
negative regulation of programmed cell death_1 **13
apoptotic signaling pathway_1 **10
regulation of apoptotic signaling pathway_2 **5
torso signaling pathway_1 *5
regulation of signaling_1 **48
regulation of response to stimulus_1 **55
cell communication **82
negative regulation of signaling **24
positive regulation of response to stimulus_1 **25
negative regulation of response to stimulus **24
signal transduction **76
regulation of cell communication_1 **49
positive regulation of cell communication_1 **22
negative regulation of cell communication **24
cell surface receptor signaling pathway **55
enzyme linked receptor protein signaling pathway **26
response to organic substance *19
cell surface receptor signaling pathway_1 **55
enzyme linked receptor protein signaling pathway_1 **26
transmembrane receptor protein tyrosine kina... **19
regulation of epidermal growth factor receptor signali... **8
epidermal growth factor receptor signaling pathway **12
heart development_1 **12
spiracle morphogenesis, open tracheal system_1 **8
hindgut morphogenesis_2 **14
cardiocyte differentiation_1 **7
periodic partitioning_1 **7
anterior/posterior pattern specification, imaginal disc_2 **5
wing disc pattern formation_3 **13
Malpighian tubule morphogenesis_2 **11
trunk segmentation_1 **6
segment polarity determination_1 *5
R7 cell differentiation_2 **24
anatomical structure morphogenesis_1 **112
sexual reproduction_2 **53
multicellular organismal reproductive process_3 **54
regulation of developmental process_1 **66
regulation of multicellular organismal process **60
tissue development_2 **89
cellular developmental process_1 **108
tube development_1 **76
system development_1 **118

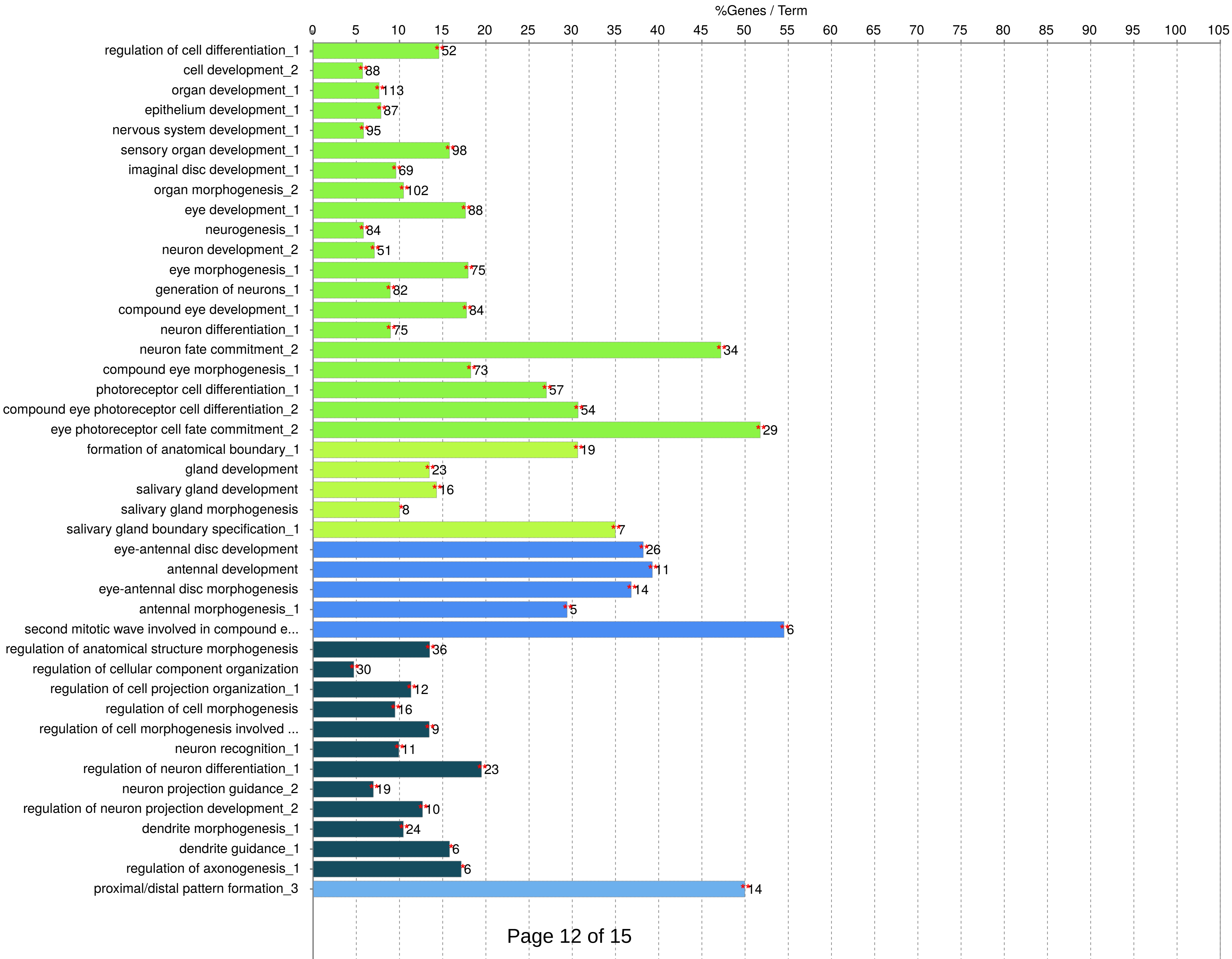

%Genes / Term
0 5 10 15 20 25 30 35 40 45 50 55 60 65 70 75 80 85 90 95 100 105
regulation of cell differentiation_1 **52
cell development_2 **88
organ development_1 **113
epithelium development_1 **87
nervous system development_1 **95
sensory organ development_1 **98
imaginal disc development_1 **69
organ morphogenesis_2 **102
eye development_1 **88
neurogenesis_1 **84
neuron development_2 **51
eye morphogenesis_1 **75
generation of neurons_1 **82
compound eye development_1 **84
neuron differentiation_1 **75
neuron fate commitment_2 **34
compound eye morphogenesis_1 **73
photoreceptor cell differentiation_1 **57
compound eye photoreceptor cell differentiation_2 **54
eye photoreceptor cell fate commitment_2 **29
formation of anatomical boundary_1 **19
gland development **23
salivary gland development **16
salivary gland morphogenesis *8
salivary gland boundary specification_1 **7
eye-antennal disc development **26
antennal development **11
eye-antennal disc morphogenesis **14
antennal morphogenesis_1 **5
second mitotic wave involved in compound e... **6
regulation of anatomical structure morphogenesis **36
regulation of cellular component organization **30
regulation of cell projection organization_1 **12
regulation of cell morphogenesis **16
regulation of cell morphogenesis involved ... **9
neuron recognition_1 **11
regulation of neuron differentiation_1 **23
neuron projection guidance_2 **19
regulation of neuron projection development_2 **10
dendrite morphogenesis_1 **24
dendrite guidance_1 *6
regulation of axonogenesis_1 *6
proximal/distal pattern formation_3 **14

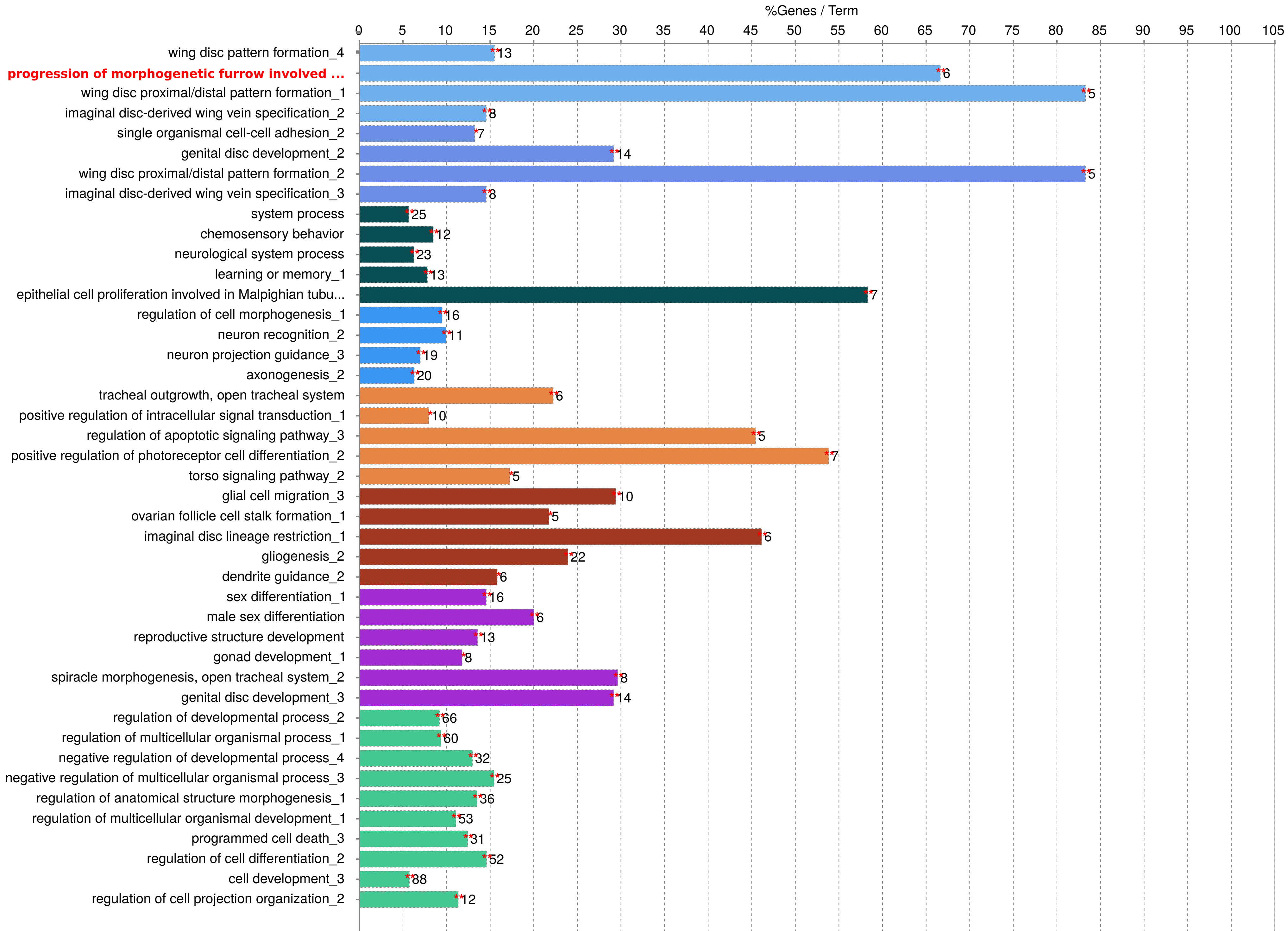

%Genes / Term
0 5 10 15 20 25 30 35 40 45 50 55 60 65 70 75 80 85 90 95 100 105
wing disc pattern formation_4 **13
progression of morphogenetic furrow involved ... **6
wing disc proximal/distal pattern formation_1 **5
imaginal disc-derived wing vein specification_2 **8
single organismal cell-cell adhesion_2 *7
genital disc development_2 **14
wing disc proximal/distal pattern formation_2 **5
imaginal disc-derived wing vein specification_3 **8
system process **25
chemosensory behavior **12
neurological system process **23
learning or memory_1 **13
epithelial cell proliferation involved in Malpighian tubu... **7
regulation of cell morphogenesis_1 **16
neuron recognition_2 **11
neuron projection guidance_3 **19
axonogenesis_2 **20
tracheal outgrowth, open tracheal system **6
positive regulation of intracellular signal transduction_1 *10
regulation of apoptotic signaling pathway_3 **5
positive regulation of photoreceptor cell differentiation_2 **7
torso signaling pathway_2 *5
glial cell migration_3 **10
ovarian follicle cell stalk formation_1 *5
imaginal disc lineage restriction_1 *6
gliogenesis_2 **22
dendrite guidance_2 *6
sex differentiation_1 **16
male sex differentiation **6
reproductive structure development **13
gonad development_1 *8
spiracle morphogenesis, open tracheal system_2 **8
genital disc development_3 **14
regulation of developmental process_2 **66
regulation of multicellular organismal process_1 **60
negative regulation of developmental process_4 **32
negative regulation of multicellular organismal process_3 **25
regulation of anatomical structure morphogenesis_1 **36
regulation of multicellular organismal development_1 **53
programmed cell death_3 **31
regulation of cell differentiation_2 **52
cell development_3 **88
regulation of cell projection organization_2 **12

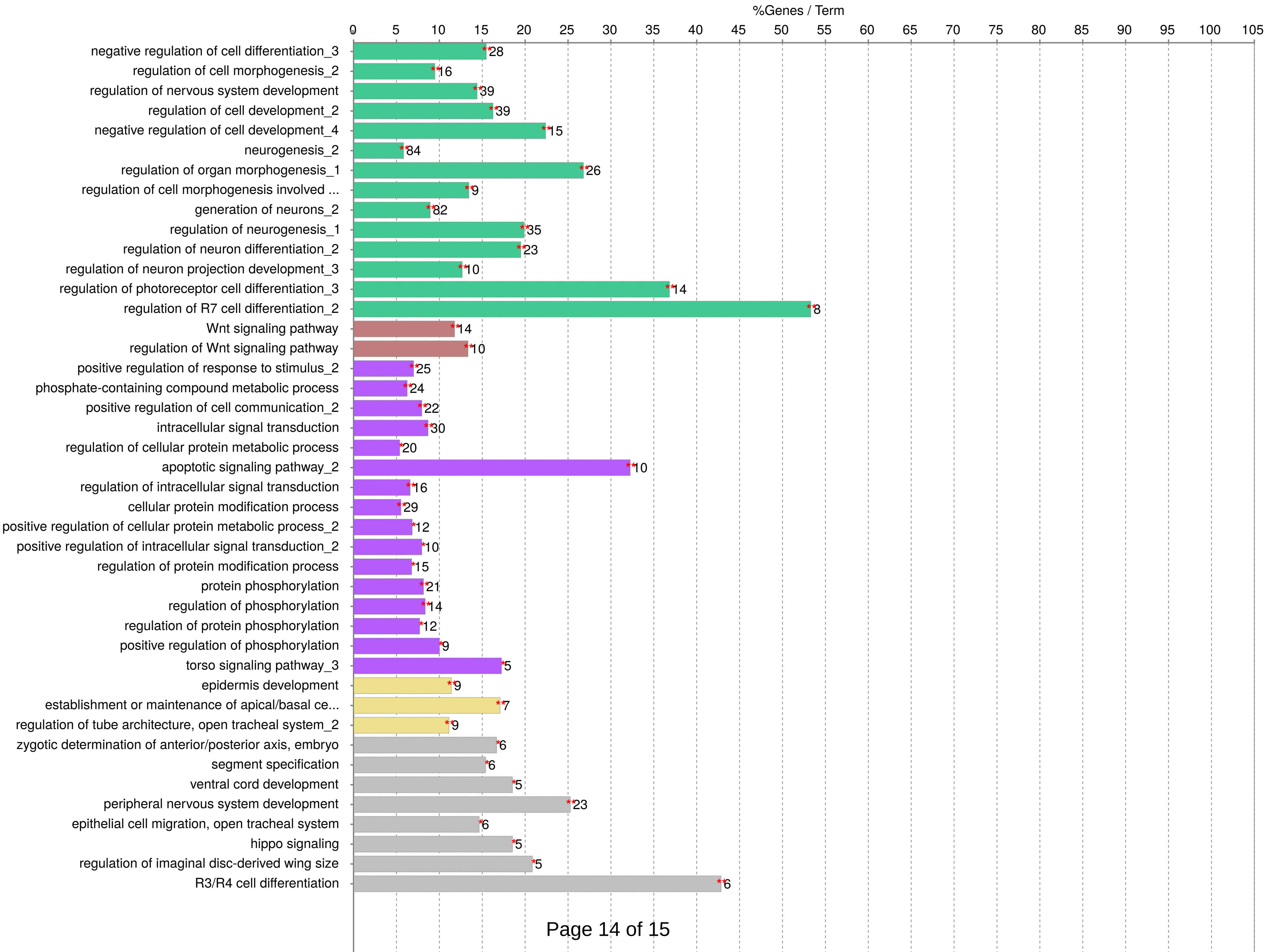
%Genes / Term
0 5 10 15 20 25 30 35 40 45 50 55 60 65 70 75 80 85 90 95 100 105
negative regulation of cell differentiation_3 **28
regulation of cell morphogenesis_2 **16
regulation of nervous system development **39
regulation of cell development_2 **39
negative regulation of cell development_4 **15
neurogenesis_2 **84
regulation of organ morphogenesis_1 **26
regulation of cell morphogenesis involved ... **9
generation of neurons_2 **82
regulation of neurogenesis_1 **35
regulation of neuron differentiation_2 **23
regulation of neuron projection development_3 **10
regulation of photoreceptor cell differentiation_3 **14
regulation of R7 cell differentiation_2 **8
Wnt signaling pathway **14
regulation of Wnt signaling pathway **10
positive regulation of response to stimulus_2 **25
phosphate-containing compound metabolic process **24
positive regulation of cell communication_2 **22
intracellular signal transduction **30
regulation of cellular protein metabolic process *20
apoptotic signaling pathway_2 **10
regulation of intracellular signal transduction **16
cellular protein modification process **29
positive regulation of cellular protein metabolic process_2 *12
positive regulation of intracellular signal transduction_2 *10
regulation of protein modification process *15
protein phosphorylation **21
regulation of phosphorylation **14
regulation of protein phosphorylation *12
positive regulation of phosphorylation *9
torso signaling pathway_3 *5
epidermis development **9
establishment or maintenance of apical/basal ce... **7
regulation of tube architecture, open tracheal system_2 **9
zygotic determination of anterior/posterior axis, embryo *6
segment specification *6
ventral cord development *5
peripheral nervous system development **23
epithelial cell migration, open tracheal system *6
hippo signaling *5
regulation of imaginal disc-derived wing size *5
R3/R4 cell differentiation **6

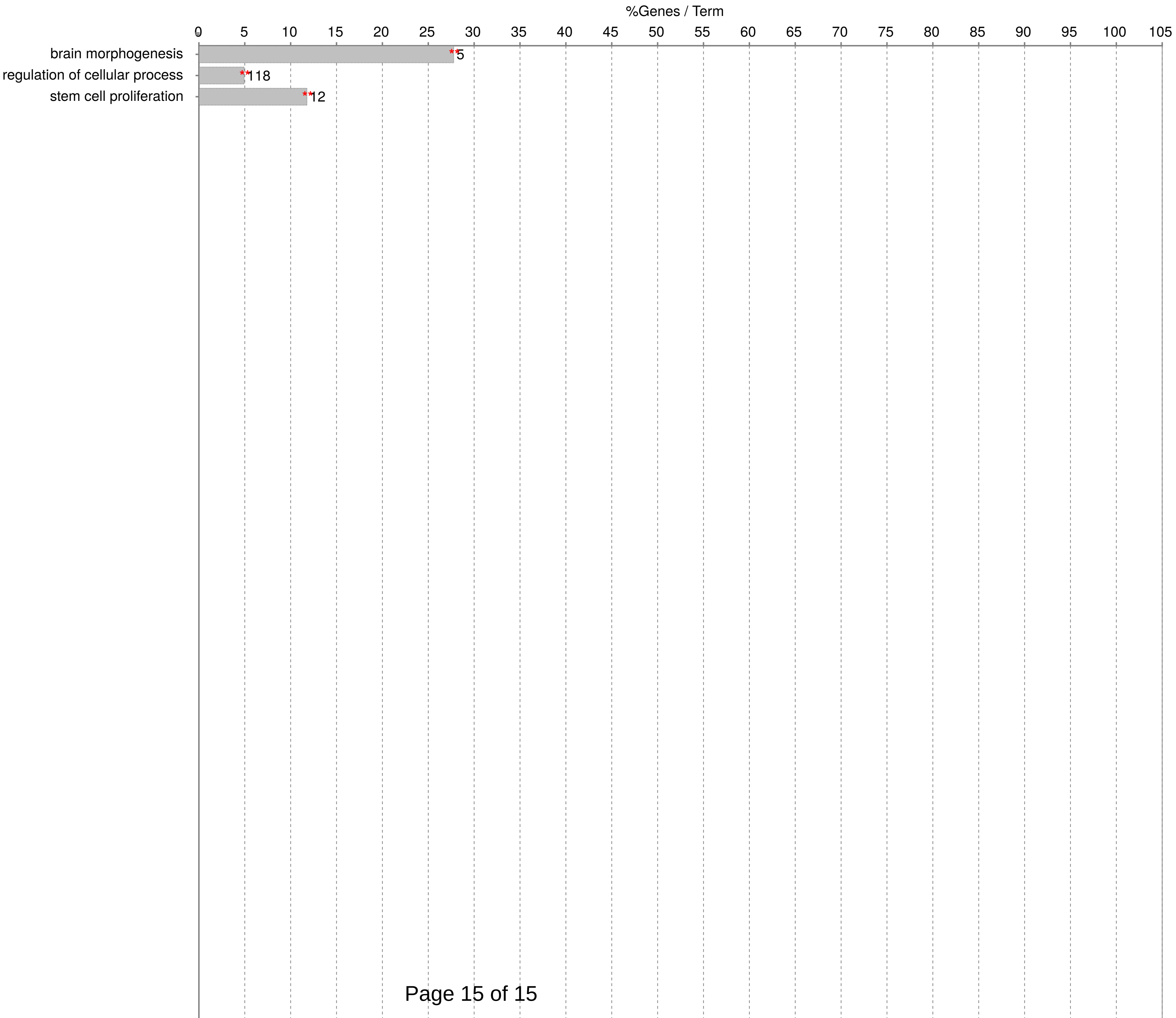
%Genes / Term
0
5
10
15
20
25
30
35
40
45
50
55
60
65
70
75
80
85
90
95
100
105
brain morphogenesis
regulation of cellular process
stem cell proliferation
**5
**118
**12
