# Supplementary material for: FlyOde - a platform for community curation and interactive visualization of dynamic gene regulatory networks in Drosophila eye development
Source: F1000Res. 2015 Dec 21;4:1484. [Version 1] doi: 10.12688/f1000research.7556.1 (PMC4786896; doi:10.12688/f1000research.7556.1)
Supplement: Supplementary file 2 [file f1000research-4-8136-s0000.tgz › 19542b53-555e-4900-8791-d12e84875cc8.docx]

# **Supplementary material**

Contents

[**Supplementary material** 1](#_Toc435707696)

[Characterization of FlyOde 1](#_Toc435707697)

[1) Content – Gene Ontology enrichment analysis 1](#_Toc435707698)

[2) Expression pattern annotation 2](#_Toc435707699)

[3) Network analysis 2](#_Toc435707700)

[Supplementary Figures and legends 3](#_Toc435707701)

[Supplementary References 5](#_Toc435707702)

## Characterization of FlyOde

### Content – Gene Ontology (GO) enrichment analysis

To characterize the content of the current FlyOde network a GO enrichment analysis was performed using the ClueGO app in Cytoscape (Bindea *et al.*, 2009; Shannon *et al.*, 2003). ClueGO identifies genes that are enriched as compared to the GO reference genome (Consortium, 2009), and groups GO terms according to the genes they share. Since FlyOde is designed to represent biological processes – currently *Drosophila melanogaster* retinal development - the corresponding GO terms were analysed, with general to medium GO levels 3-8 (with level 20 being the most specific) and a relatively low kappa score threshold of 0.45 to obtain larger groups and to reduce the number of ungrouped terms. To avoid redundancy GO term fusion was applied. Since FlyOde contains only manually curated confirmed data, the annotation evidence was chosen relatively stringent with “inferred from experiment”, “by curator”, “from direct assay”, “from genetic interaction”, “from mutant genotype”, and “traceable author statement”. Only terms with a p value smaller or equal to 0.05 were chosen. Right sided hypergeometric test and Bonferroni correction were applied. With these settings 331 GO terms were found to be enriched in FlyOde and partitioned into 57 groups and 11 ungrouped terms (Supplementary Figure 1).

As expected, enriched terms include those related to general development and morphogenesis, pattern formation and polarity, apoptosis, mitosis, and regulation of transcription. 80 of the currently 144 FlyOde genes are annotated with eye development, which corresponds to 18 % of the reference genes annotated with this term. Since FlyOde consists mostly of genes involved in retinal development (except for the six Rhodopsins), about 44 % of FlyOde genes are not included in this annotation by the Gene Ontology Consortium. The enrichment of more specific terms like neuron fate specification (86 % of the GO reference, corresponding to 12 FlyOde genes), eye photoreceptor cell fate commitment (52 %, 29 genes), photoreceptor cell differentiation (26 %, 57 genes), progression of morphogenetic furrow involved in compound eye morphogenesis (66 %, 6 genes), R7 cell development (38 %, 8 genes), R7 cell differentiation (42 %, 24 genes), R8 cell differentiation (83 %, 15 genes) and R8 cell fate specification (100 %, 7 genes) indicates that the major pathways are covered by FlyOde, but also that more data have to be collected. Genes involved in retinal development (as inferred from FlyOde) are shared with processes in other organs like leg disc development (19 %, 24 genes), sex differentiation (14 %, 16 genes), haemocyte differentiation (29 %, 13 genes), epithelial cells, Malpighian tubule, trachea formation, and development of salivary gland and antenna (no numbers are given for the latter processes, since they are represented by several terms). It will be interesting to compare their developmental networks in the future.

Since the low number of enriched genes in some processes, e.g. progression of morphogenetic furrow (in total 9 GO annotated genes), indicates incomplete GO annotation, this analysis reflects biology only partially and also highlights the need for more thorough curation, which FlyOde is aiming to achieve via its community-driven effort.

A complementary analysis was performed with the enrichment analysis tool at the Gene Ontology Consortium homepage (<http://geneontology.org/>) followed by use of REVIGO (<http://revigo.irb.hr/>)(Supek *et al.*, 2011) to remove redundant terms and represent the result as a treemap (Supplementary Figure 2). This led to similar results as analysis with ClueGO.

### Expression pattern annotation

The information on expression patterns that can be obtained by FlyOde was compared with the most comprehensive database for *Drosophila* research, FlyBase (Dos Santos et al., 2015). Random samples were investigated using the Flybase QueryBuilder tool (as of June 2015) for expression patterns and the Cytoscape search tool for FlyOde, which corresponds to the online filter function. Filtering for general terms like PR, anterior and posterior of morphogenetic furrow, and pupa PR, both tools are mostly comparable, with FlyBase QueryBuilder being partially superior (third instar larva PR) (Supplementary Figure 3). However, as the definition becomes narrower FlyOde gives more hits than the FlyBase QueryBuilder (third instar larva R3, late third instar larva PR, pupa R3 and R7, pupa DRA R8). This shows that FlyOde already stands out in defining expression patterns during PR differentiation when combining annotation according to specific cell type and developmental stage. In the adult, when differentiation has mostly been accomplished, the performance of FlyOde and FlyBase QueryBuilder are again comparable. Importantly, with FlyOde various filters can be combined with Boolean operators to exclude genes expressed in specific cell types and developmental stages.

### Network analysis

In FlyOde the number of protein-DNA interactions driving development appears to be much higher than that of protein-protein interactions (73 and 39, respectively)*.* This could be due to developmental mechanisms that determine cell types with increasing specificity by initiating expression of specific transcription factors in cascades rather than by protein modifications. It might however simply reflect the state of published research, which produces more data (as recorded for our analysis, e.g. excluding evidence that is solely derived from high throughput data) for protein-DNA than for protein-protein interactions due to technical reasons. Additionally, the high number of unassigned (genetic, indirect (150)) interactions, for which no specific mechanism has been established, has to be taken into account. This finding highlights the gaps in our current knowledge that could represent an entry point for future research. Discrimination of redundant interactions versus true feed-forward loops would be one possible approach. For example, the negative and positive genetic interactions of warts with Rh5 and Rh6, respectively, might be explained by the confirmed interaction of warts with yorkie, which directly activates Rh5 and inhibits Rh6 expression (Supplementary Table, Supplementary Figure 4a).

The relatively high number of ato targets (15) reflects its role as a proneural master regulator, but is also due to inclusion of results from the paper of Potier et al., (Potier et al., 2014), where sophisticated bioinformatic analysis, expression data, and enhancer-reporter fly lines were used (13 ato targets). These interactions are annotated with the prefix “icistarget+_” in the edge attribute interaction type.

The FlyOde network was manually analysed for its motif content (Alon, 2007). Positive autoregulation tends to increase cell–cell variability, thus leading to different cell populations. It can also act to maintain gene expression even after the initial stimulus is gone. Both features are applied during cell differentiation, which is driven by ato, da, and so by enhancing their own expression (Supplementary Figure 4b).

Negative autoregulation can reduce cell–cell variation in protein levels, thus keeping the cells of a precursor organ in a similar state and double negative feedback loops can act as toggle switches between the two states.

Tsh and tio apply both mechanisms by directly inhibiting their own and each other’s activity (Supplementary Figure 4c). Tio and tsh are expressed anterior to the morphogenetic furrow, where cells have not started differentiation yet and activate homothorax, a major negative regulator of morphogenetic furrow progression and eye development.

Composite feedback loops are more common than pure protein-DNA feedback loops due to enhanced stability, which results from the faster feedback via protein-protein interactions and are applied here in positive as well as negative feedback loops.

The incoherent feed forward loop (iFFL) including oc, dve, and Rh3 most likely acts to normalize noise of oc (Supplementary Figure 4d) (Johnston et al., 2011). Additionally, iFFLs have been implicated in temporal pulse responses, response acceleration, and dose-dependent biphasic responses.

For the final output of this network, which culminates in Rh expression, multi-output signalling of several inputs is combined to give a variation of dense overlapping regulons (Supplementary Figure 4e). The R8 marker Sens inhibits expression of Rh3 and activates Rh6 (Xie et al., 2007). Dve inhibits expression of Rh3, Rh5, and Rh6 in outer PRs and yR7s (Johnston et al., 2011). Oc is expressed in all PRs and directly activates Rh3 and at the same time inhibits Rh3 expression through activation of Dve (see incoherent ffd above), activates Rh5 expression, and has been shown to activate and to inhibit Rh6 expression. Oc also activates melted expression, which directly activates pale R8-specific yorkie to activate Rh5 and inhibit Rh6 expression.

Thus, the basic motifs, that have been described to be involved in developmental networks are also found in FlyOde (Supplementary Table) (Alon, 2007; Davidson, 2010).⁠

## Supplementary Figures and legends

**Supplementary Figure 1**: see separate file. ClueGO results. The bar graph displays the percentage of FlyOde genes for each term relative to the reference. The absolute numbers of the respective FlyOde genes are shown next to the bars. Colours indicate term groups shared by genes enriched in FlyOde. Specific terms mentioned in the text are highlighted in red.


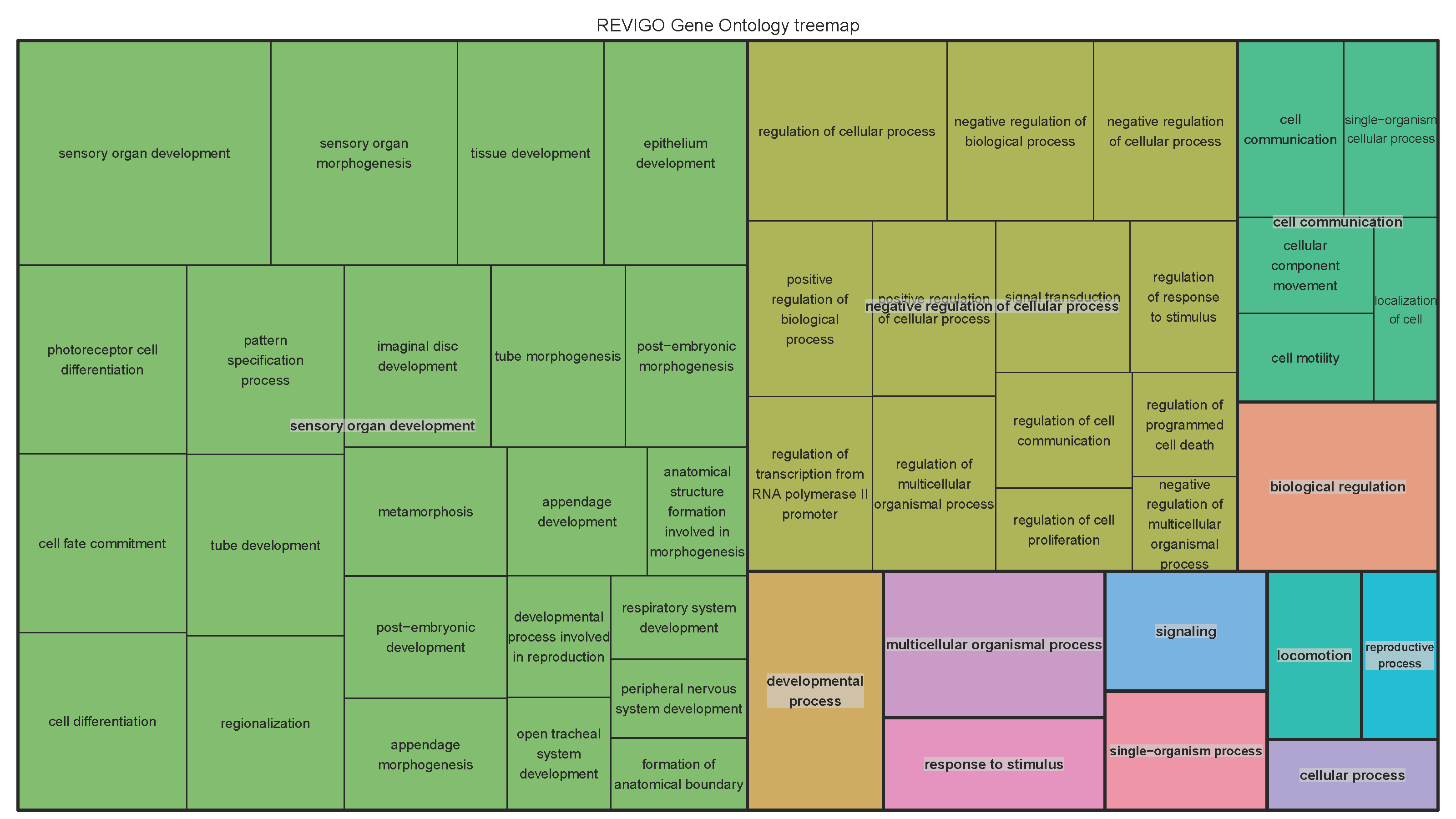


**Supplementary Figure 2**: Treemap of GO terms enriched in FlyOde. Enriched GO terms were determined with the GO-term enrichment analysis tool at the GO Consortium homepage. Using REVIGO redundant terms were removed and the remaining ones were visualized.


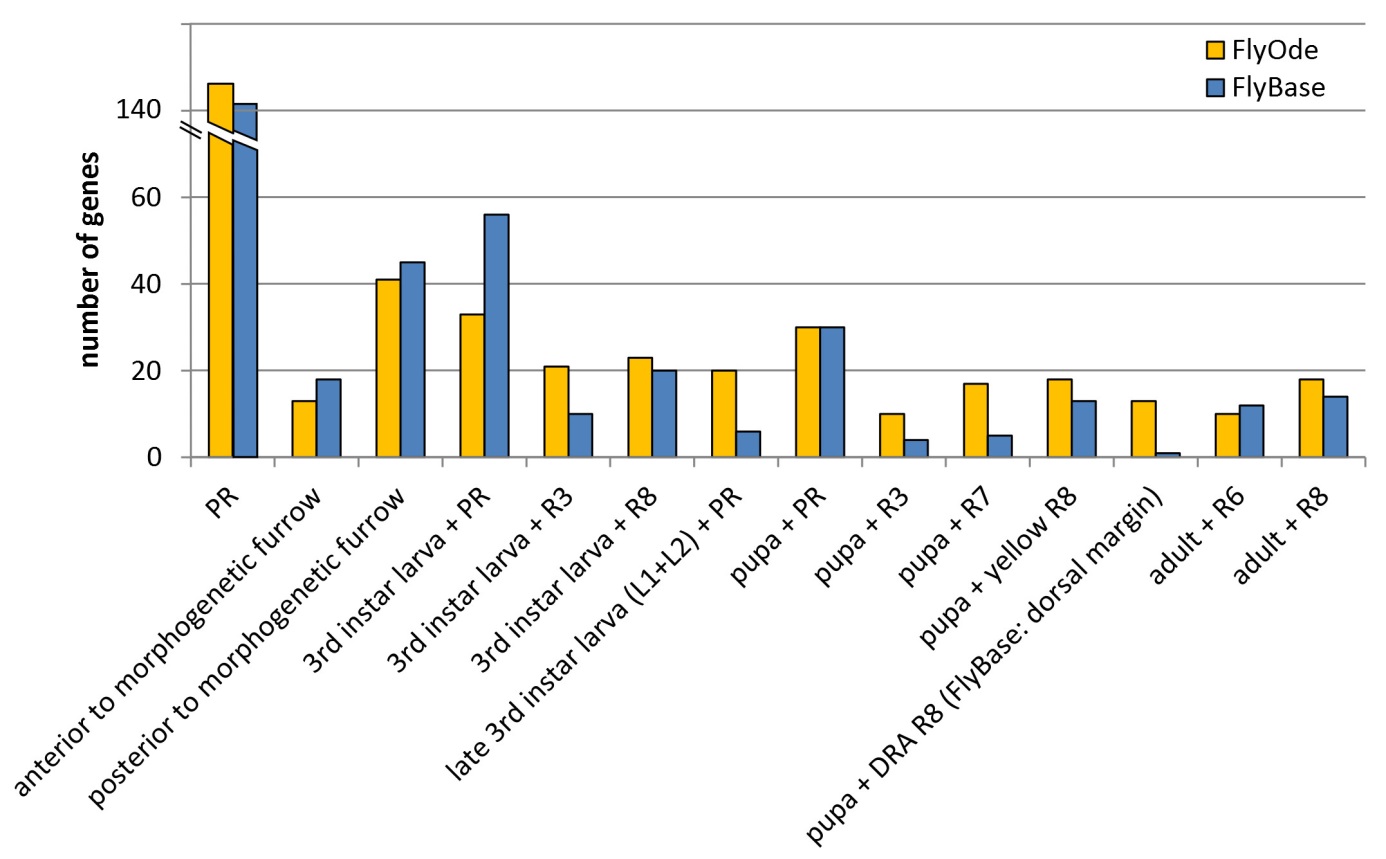


**Supplementary Figure 3:** The number of genes obtained for indicated expression pattern term combinations with FlyOde and the FlyBase QueryBuilder are shown.


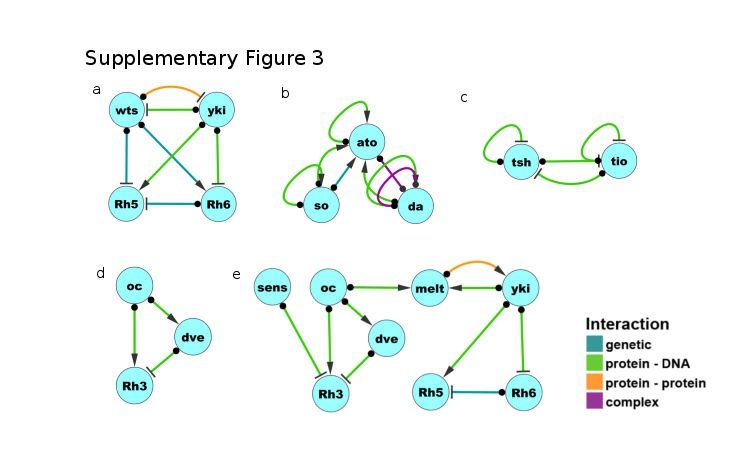


**Supplementary Figure 4**: Network motif examples represented in FlyOde. For further explanation see Supplementary text, section on network analysis.

|  |  | **Protein-DNA** | **Protein-Protein** | **Composite** |
| --- | --- | --- | --- | --- |
| **Autoregulation** | Negative | Tio, tsh | - | N/A |
|  | Positive | ato, da, so | stan |  |
| **Feedback loop** | Negative | tsh – tio | - | edl - aop,  mir-7 – aop, yorkie - warts |
|  | Positive | - | - | Yorkie - melted |
| **Feed-forward loop (incoherent)** | N/A | oc pd_pos dve, dve pd_neg Rh3, oc pd_pos Rh3 | - | - |
| **Supplementary Table**: Network motif summary in FlyOde. Prior to this analysis indirect (genetic) interactions were removed to restrict identification to minimal motifs. The number of motifs increases strongly when genetic interactions are included. | | | | |

##

## Supplementary References

Alon,U. (2007) Network motifs: theory and experimental approaches. *Nat. Rev. Genet.*, **8**, 450–461.

Bindea,G. *et al.* (2009) ClueGO: a Cytoscape plug-in to decipher functionally grouped gene ontology and pathway annotation networks. *Bioinformatics*, **25**, 1091–3.

Consortium,T.R.G.G. of the G.O. (2009) The Gene Ontology’s Reference Genome Project: a unified framework for functional annotation across species. *PLoS Comput. Biol.*, **5**, e1000431.

Davidson,E.H. (2010) Gene Regulatory Networks In Development And Evolution Academic Press.

Johnston,R.J. *et al.* (2011) Interlocked Feedforward Loops Control Cell-Type-Specific Rhodopsin Expression in the Drosophila Eye. *Cell*, **145**, 956–968.

Potier,D. *et al.* (2014) Mapping Gene Regulatory Networks in Drosophila Eye Development by Large-Scale Transcriptome Perturbations and Motif Inference. *Cell Rep.*, **9**, 2290–2303.

Dos Santos,G. *et al.* (2015) FlyBase: introduction of the Drosophila melanogaster Release 6 reference genome assembly and large-scale migration of genome annotations. *Nucleic Acids Res.*, **43**, D690–7.

Shannon,P. *et al.* (2003) Cytoscape: a software environment for integrated models of biomolecular interaction networks. *Genome Res.*, **13**, 2498–504.

Supek,F. *et al.* (2011) REVIGO summarizes and visualizes long lists of gene ontology terms. *PLoS One*, **6**, e21800.

Xie,B. *et al.* (2007) Senseless functions as a molecular switch for color photoreceptor differentiation in Drosophila. *Development*, **134**, 4243–53.
