# Supplementary material for: FlyOde - a platform for community curation and interactive visualization of dynamic gene regulatory networks in Drosophila eye development
Source: F1000Res. 2015 Dec 21;4:1484. [Version 1] doi: 10.12688/f1000research.7556.1 (PMC4786896; doi:10.12688/f1000research.7556.1)
Supplement: Peer review history

# Peer review: Referee response for version 1

**DOI:** 10.5256/f1000research.8136.r11677

**Published:** 2016-02-15

**Authors:** Fernando Casares

**Affiliations:** 1 CABD (Andalusian Centre for Developmental Biology), CSIC and Pablo de Olavide University, Seville, Spain

The Drosophila eye gene regulatory network ranks among the best textbook examples of organogenetic GRNs. FlyODE, by Koestler and colleagues is a welcomed attempt at integrating the many bits of expression, regulation, molecular interactions and functional analyses obtained to date, by providing a cytoscape-like tool in the form of a curated, directed graph network. FlyODE’s also allows the expansion of this network through community-based contributions –which is a great philosophy. Therefore FlyODE is a valuable tool for those working on gene networks in general and on fly eyes in particular, and will help in expanding the network (and our knowledge of the system) further. FlyODE includes references to spatial gene expression patterns. It would be good if the authors tried to unify the somehow ambiguous terms used by different labs, such as “preproneural/precursors/anterior to the MF” or “proneural/immediately anterior to the MF”, or perhaps provide some “equivalence” terms. When sorting genes according to their expression along third instar, I found that the network includes more genes at early L3 than at mid-L3, which shouldn’t be the case –as the process unfolds more genes are called into action.

I have read this submission. I believe that I have an appropriate level of expertise to confirm that it is of an acceptable scientific standard.

# Response

**Published:** 2016-02-15

**Authors:** Stefan A. Koestler

**Affiliations:** Bogazici University, Turkey

Thank you very much for your feedback!

We are working on improving the ontology, e.g. by adjusting it to the Gene Ontology, and will incorporate your suggestions.

The layout is currently done manually and is certainly to some extent biased depending on the references used for annotation. This can be expected to improve with an increasing number of annotations/references. In addition, we are planning an automatic layout to calculate the position from the annotations. In any case one has to keep in mind that it will only indicate a single (mean) value, while a gene might display a more complex expression pattern. Thus, currently one has to look at the report panel below the network for more detailed information. We are thinking about adding a graph displaying the expression profile when hovering over a node to make this information more intuitively accessible.

# Peer review: Referee response for version 1

**DOI:** 10.5256/f1000research.8136.r12188

**Published:** 2016-02-12

**Authors:** Bassem A Hassan

**Affiliations:** 1 Center for the Biology of Disease, VIB and University of Leuven School of Medicine, Leuven, Belgium; 2 Institut du Cerveau et de la Moelle epiniere (ICM), IHU Pitié Salpétrière, Paris, France

This is a very nice and useful tool to quickly and efficiently access information about fly retina development. The fact that it can be extended to any tissue and is an open platform for community-based annotation makes it all the more useful. I applaud the authors for their efforts and thank them for making such a cool and intuitive tool available. The video tutorial is very nice and helpful as well.

I have read this submission. I believe that I have an appropriate level of expertise to confirm that it is of an acceptable scientific standard.

# Peer review: Referee response for version 1

**DOI:** 10.5256/f1000research.8136.r12186

**Published:** 2016-02-11

**Authors:** Pelin Cayirlioglu Volkan

**Affiliations:** 1 Department of Biology, Duke University, Durham, NC, USA

FlyOde is a very nice resource for labs, which are interested in visualizing developmental dynamics of gene regulatory networks required for the development for many neuronal types in the Drosophila eye, in addition to finding lineage specific markers. It will be valuable in the future to expand this to other sensory systems such as the olfactory system.  The title and the abstract are appropriate. The video presentation is very useful.

I have read this submission. I believe that I have an appropriate level of expertise to confirm that it is of an acceptable scientific standard.
